# Sixty years of plant community change in Europe indicate a shift toward nutrient-richer and denser vegetation

**DOI:** 10.1126/sciadv.aeb2493

**Published:** 2026-04-10

**Authors:** Gabriele Midolo, Adam Thomas Clark, Milan Chytrý, Franz Essl, Stefan Dullinger, Ute Jandt, Helge Bruelheide, Jürgen Dengler, Irena Axmanová, Svetlana Aćić, Olivier Argagnon, Idoia Biurrun, Gianmaria Bonari, Alessandro Chiarucci, Renata Ćušterevska, Pieter De Frenne, Michele De Sanctis, Jan Divíšek, Jiří Doležal, Tetiana Dziuba, Rasmus Ejrnæs, Emmanuel Garbolino, Anke Jentsch, Borja Jiménez-Alfaro, Jonathan Lenoir, Jesper Erenskjold Moeslund, Francesca Napoleone, Sabine B. Rumpf, Jens-Christian Svenning, Grzegorz Swacha, Irina Tatarenko, Martin Večeřa, Denys Vynokurov, Petr Keil

**Affiliations:** ^1^ Department of Spatial Sciences, Faculty of Environmental Sciences, Czech University of Life Sciences Prague, Praha-Suchdol, Czech Republic.; ^2^ Department of Biology, University of Graz, Graz, Austria.; ^3^ Department of Botany and Zoology, Faculty of Science, Masaryk University, Brno, Czech Republic.; ^4^ Division of BioInvasions, Global Change and Macroecology, Department of Botany and Biodiversity Research, University of Vienna, Vienna, Austria.; ^5^ Division of Biodiversity Dynamics and Conservation, Department of Botany and Biodiversity Research, University of Vienna, Vienna, Austria.; ^6^ Institute of Biology/Geobotany and Botanical Garden, Martin Luther University Halle-Wittenberg, Halle, Germany.; ^7^ German Centre for Integrative Biodiversity Research (iDiv) Halle-Jena-Leipzig, Leipzig, Germany.; ^8^ Vegetation Ecology Research Group, Institute of Natural Resource Sciences (IUNR), Zurich University of Applied Sciences (ZHAW), Wädenswil, Switzerland.; ^9^ Bayreuth Center of Ecology and Environmental Research (BayCEER), University of Bayreuth, Bayreuth, Germany.; ^10^ Department of Botany, Faculty of Agriculture, University of Belgrade, Serbia.; ^11^ Conservatoire Botanique National Méditerranéen, Hyères, France.; ^12^ Department of Plant Biology and Ecology, Faculty of Science and Technology, University of the Basque Country UPV/EHU, Bilbao, Spain.; ^13^ Department of Life Sciences, University of Siena, Siena, Italy.; ^14^ National Biodiversity Future Center (NBFC), Palermo, Italy.; ^15^ BIOME Lab, Department of Biological, Geological, and Environmental Sciences, Alma Mater Studiorum, University of Bologna, Bologna, Italy.; ^16^ Faculty of Natural Sciences and Mathematics, Ss. Cyril and Methodius University, Skopje, North Macedonia.; ^17^ Forest and Nature Lab, Faculty of Bioscience Engineering, Ghent University, Gontrode, Belgium.; ^18^ Department of Environmental Biology, Sapienza University of Rome, Rome, Italy.; ^19^ Institute of Botany of the Czech Academy of Sciences, Třeboň, Czech Republic.; ^20^ Department of Geobotany and Ecology, M.G. Kholodny Institute of Botany, National Academy of Sciences of Ukraine, Kyiv, Ukraine.; ^21^ Department of Ecoscience, Aarhus University, Aarhus, Denmark.; ^22^ ISIGE, MINES Paris PSL, Fontainebleau, France.; ^23^ Disturbance Ecology and Vegetation Dynamics, University of Bayreuth, Bayreuth, Germany.; ^24^ Biodiversity Research Institute (IMIB), University of Oviedo-CSIC-Principality of Asturias, Oviedo, Spain.; ^25^ Department of Organismal and Systems Biology, University of Oviedo, Oviedo, Spain.; ^26^ UMR CNRS 7058 Ecologie et Dynamique des Systèmes Anthropisés (EDYSAN), Université de Picardie Jules Verne, Amiens, France.; ^27^ Department of Environmental Sciences, University of Basel, Basel, Switzerland.; ^28^ Center for Ecological Dynamics in a Novel Biosphere (ECONOVO), Department of Biology, Aarhus University, Aarhus, Denmark.; ^29^ Botanical Garden, University of Wrocław, Wrocław, Poland.; ^30^ School of Environment, Earth and Ecosystem Sciences, Open University, Milton Keynes, UK.

## Abstract

Anthropogenic impacts are reshaping plant biodiversity patterns, yet how community-composition shifts track environmental change at large spatial and temporal scales remains unclear. Here, we quantified trends in community-mean plant ecological indicator values (light, temperature, soil moisture, soil nitrogen, and soil reaction) across European vegetation between 1960 and 2020. We used spatiotemporal interpolation based on 644,524 plots and analyzed 18,345 time series encompassing diverse habitats. We found a clear shift in community composition over the past six decades with a steep increase in nitrogen-demanding species across all main habitat types, accompanied by a moderate increase in shade-tolerant species. Forest communities shifted toward species associated with higher soil pH, while wetland communities showed a decline in moisture-dependent species over time. Conversely, temperature indicator values were largely stable, except for recent thermophilization in alpine habitats. Our results indicate a widespread trend toward denser vegetation driven by eutrophication and changes in management practices.

## Teaser

Bioindication reveals a 60-year shift toward denser, more productive vegetation, but a weak thermophilization signal.

## INTRODUCTION

Human activities have directly or indirectly exposed biodiversity to multiple threats, such as climate change and resource exploitation in various parts of the world (*1*). However, studies focusing on alpha diversity (i.e., local species richness) often reveal no net gain or loss over time across different habitat types (*2*–*6*). By contrast, substantial evidence indicates a pronounced temporal turnover in community composition across various ecosystems globally (*3*, *7*), and specifically in European plant communities (*8*–*11*). There is well-established evidence that environmental pressures driving these changes include modifications of hydrological regimes (*9*, *12*), nutrient enrichment (specifically nitrogen and phosphorous) (*8*, *13*, *14*), global warming (*10*, *15*, *16*), alterations in disturbance regimes affecting resources and light availability (*9*, *14*, *17*), and changes in soil pH (*17*–*19*).

The various environmental pressures driving community change can be revealed through shifts in the prevalence of “indicator species,” i.e., species whose presence is indicative of certain ecological conditions (*10*, *15*, *20*, *21*). Such a concept is known as “bioindication” (*22*). Ecological indicator values (EIVs) constitute a well-established expert-based numerical system widely applied for this purpose in Europe (*23*–*25*). EIVs classify species based on their mean realized niche position, allowing inference of environmental conditions by averaging the EIVs of co-occurring species at a site (*22*). Despite EIVs having limitations, such as being frequently correlated and capturing environmental conditions only indirectly, they enable estimates of ecological dimensions where direct measurements are missing. Thus, temporal shifts in species composition within a community enable us to monitor these changes through the lens of bioindication, providing insights into the underlying shifts in community-level ecological preferences. For instance, an increasing proportion of warm-adapted species is interpreted as a sign of thermophilization driven by global warming (*15*, *20*). As another example, changes in management practices, such as the cessation of grazing or coppicing (*10*, *14*, *26*), often result in an increasing proportion of shade-tolerant species and hence a decrease in the community mean of EIVs for light conditions.

Broad spatiotemporal changes in environmental conditions can thus be reconstructed using bioindication and large-scale vegetation survey data (*18*). Because the fine-grained reconstruction of past environmental conditions is often impossible, assessing temporal changes in EIVs is especially useful in time series data (*21*). In addition, EIVs are generally robust to observer-related errors during vegetation resurveys (*27*). Despite such advantages, large-scale assessments of temporal changes in EIVs are limited by the availability of comprehensive time series of vegetation resurveys. Such data are available in Europe (*28*), but they are patchy, both spatially and temporally. Thus, we still lack a broad-scale and fine-resolution assessment of how environmental conditions, as indicated by plant community composition, have changed over time across Europe.

To fill this knowledge gap, we here applied a spatiotemporal interpolation (*6*) using single-survey vegetation-plot data, the most widely available type of data in European vegetation databases (*29*). In our approach, we treated the community-mean EIVs (CM_EIV_s) from each one-time survey plot as a single observation in space and time. These observations were then used to fill gaps in the “space-time cube” (*30*) using machine learning, allowing us to reconstruct their temporal dynamics through model predictions (*6*). This is feasible due to the inherent spatial and temporal autocorrelation in species occurrences and the environment (*31*), a feature that tree-based ensemble models can effectively exploit to capture complex interactions and boost the accuracy of site-level predictions.

We aimed to reconstruct how plot-level CM_EIV_s have changed across Europe and within major habitat types (forests, grasslands, scrub, and wetlands) over the past six decades (1960–2020), enabling comparisons of the direction and magnitude of change. We combined a consensus dataset of European EIV systems for light, temperature, and soil variables (moisture, nitrogen, and reaction), available for 13,874 vascular plant taxa (*25*), and calculated CM_EIV_s as the average EIVs of species occurring in each of the 644,524 vegetation-plot records from the European Vegetation Archive (EVA) (*29*) and ReSurveyEurope (*28*). We interpolated CM_EIV_s for each indicator using random forests (*32*, *33*) as a function of space (longitude, latitude, and elevation), time, plot size, and species richness. We also analyzed CM_EIV_ changes over the same period across 57,255 observation records from 18,345 resurvey plots available in ReSurveyEurope and used these data to independently validate our interpolation approach. In Table 1, we detail the expected shifts in EIVs at the community level. We hypothesized these changes to be influenced by various abiotic and biotic characteristics of European habitat types, causing habitat-dependent trends (*10*, *34*, *35*). We built our expectations based on several key putative drivers affecting plant community changes, specifically global warming, environmental pollution, and land-use change (Table 1).

**Table 1.** Hypothesized directions of temporal changes in EIVs in European plant communities. The sign of the expected change indicates an increase (+) or decrease (–) in EIVs. The table reports the related putative drivers and the ecological processes (species composition changes) through which we expect these drivers to affect CM_EIV_s.

EIV	Expected change over time	Affected ecosystems	Putative drivers	Ecological processes
**Light**	+	Forests	Increased overstory disturbance events (e.g., windthrows, drought, and bark beetle outbreaks)	Gains in light-demanding species following canopy openness due to enhanced tree mortality (*16*, *76*)
	–	All	Management cessation; nutrient pollution; CO_2_ fertilization	Gains in species adapted to shadier vegetation and higher above-ground biomass (*9*, *35*, *37*, *51*)
**Temperature**	+	Cold and temperate	Global warming	Losses of cold-adapted species and gains in thermophilous species (*10*, *15*, *16*, *20*)
	–	Temperate and warm, especially forests	Management cessation; nutrient pollution; CO_2_ fertilization	Losses of thermophilous species following cooler microclimatic conditions induced by increased canopy and/or herb-layer density (*14*)
**Moisture**	+	Dry	Management cessation	Losses of dry-habitat specialists and gains of mesophilous species (*10*, *14*, *26*, *77*)
	–	Wet	Increasing drought frequency and land-use change (e.g., soil drainage)	Losses of hygrophilous (wet-adapted) species following lowered water tables (*9*, *12*, *78*)
**Nitrogen**	+	All, especially nutrient-poor	Management cessation; nutrient pollution	Losses of oligotrophic, weakly competitive species and gains of competitive, nutrient-tolerant species following biomass and litter build-up and nutrient enrichment (*9*–*11*, *37*)
**Reaction**	+	All	Abatement of ammonia and sulfur emissions (reduced “acid rain”) after 1980s	Gains in less acidophilous species in response to soil pH recovery (*18*, *51*, *79*, *80*)
	–	All	Ammonia and sulfur emissions (1960–1980s)	Gains in acidophilous species in response to soil acidification caused mainly by sulfur deposition (*17*, *19*)

## RESULTS

**Fig. 1.**
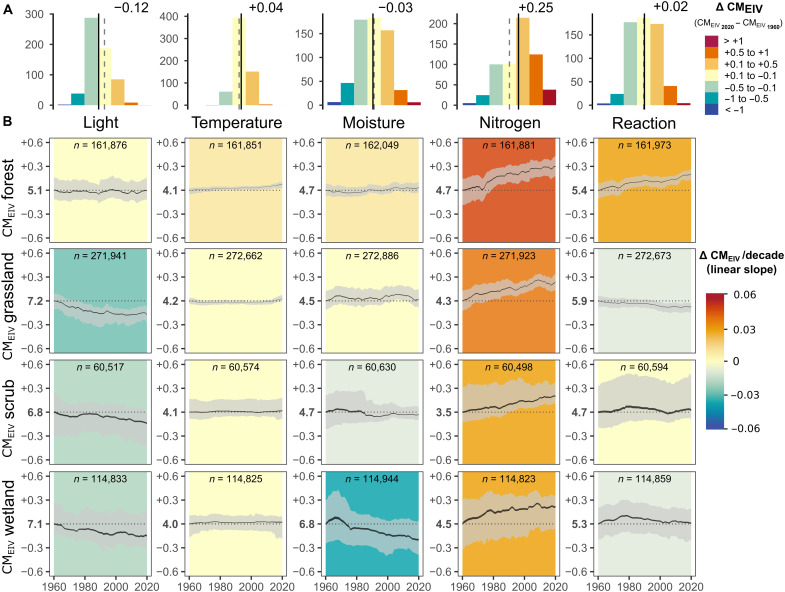
Interpolated temporal trends of CM_EIV_ across 610,537 European vegetation plots sampled between 1960 and 2020. Histograms in (**A**) show the distribution of CM_EIV_ changes for the year 2020 compared to 1960; each histogram reports the values of the average CM_EIV_ change across plots (vertical solid line) relative to zero change (vertical dashed line). (**B**) Temporal trends in average CM_EIV_s, calculated as the mean CM_EIV_ across plots per year and habitat for each of the 500 decision trees within the random forest model. Trends are summarized as a 95% confidence interval (CI) around the mean (black ribbon) and a prediction interval between the 0.025 and 0.975 quantiles (gray ribbon). Linear regression slopes (background colors) are fitted on the mean predicted values of CM_EIV_ across all plots for each year. The *y*-axis scale is standardized to the baseline average CM_EIV_ estimated for the year 1960 and displayed in bold. CM_EIV_s are interpolated using a fixed plot size (225 m^2^ for forests, 16 m^2^ for grasslands, 50 m^2^ for scrub, and 25 m^2^ for wetlands).

**Fig. 2.**
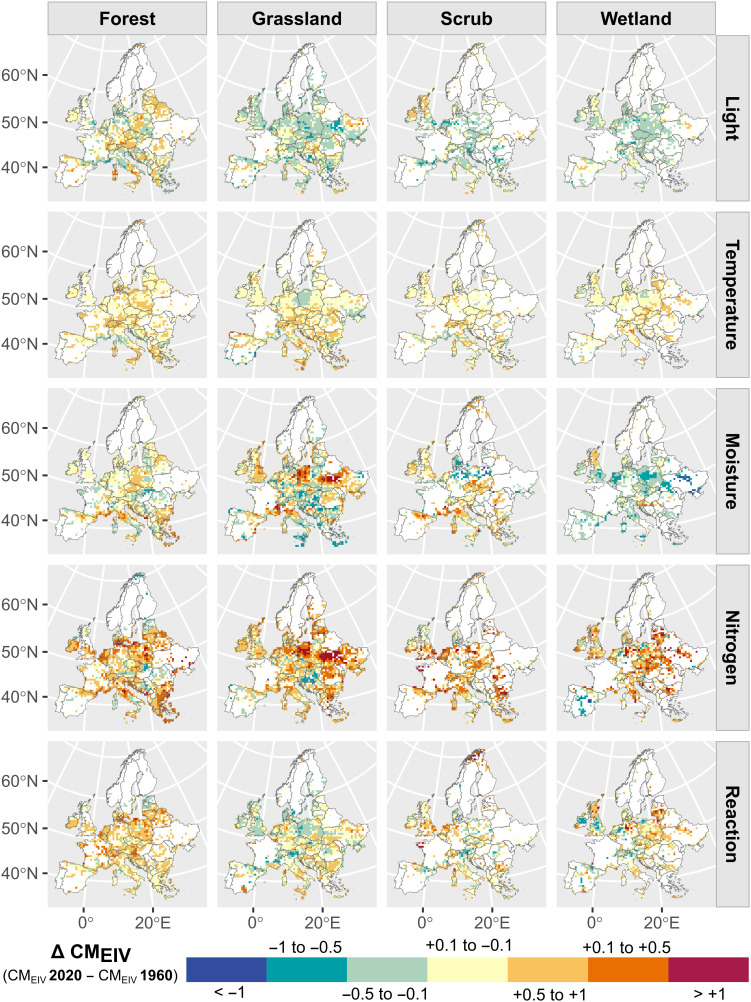
Geographic patterns in the temporal trends of CM_EIV_s from 1960 to 2020 across main European habitat types. The maps show the average change in plot-level CM_EIV_ for each EIV variable, aggregated across all plots found within 50-km–by–50-km grid cells. CM_EIV_ values are interpolated using predictions across all plots for the years 1960 and 2020, using a fixed plot size that is representative of the focal habitat (225 m^2^ for forests, 16 m^2^ for grasslands, 50 m^2^ for scrub, and 25 m^2^ for wetlands). Only grid cells containing at least five plots per habitat type sampled within this period are shown. An interactive map at finer resolution is available at http://gmidolo.shinyapps.io/interpolated_EIV_change_app/.

**Fig. 3.**
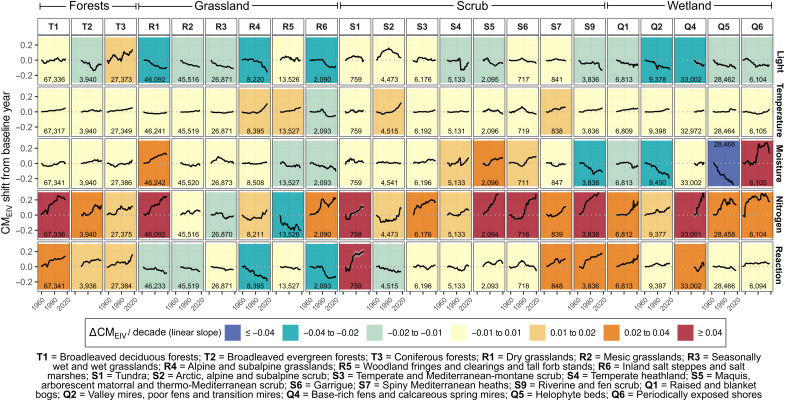
Interpolated changes in CM_EIV_s across EUNIS level 2 habitats. Each panel shows temporal trends in average CM_EIV_s, calculated as the mean CM_EIV_ across plots for each year and habitat type. Linear regression slopes (background colors) express the mean predicted changes in CM_EIV_ every 10 years. The *y*-axis is standardized to the baseline average CM_EIV_ estimated for the first year of interpolation. To ensure robust temporal coverage and limit extrapolation, interpolated time series for each habitat were restricted to plots sampled between the 0.05 and 0.95 quantiles of sampling years, using a fixed plot size (the habitat-specific median). Estimates are based on models trained on a subset of 384,254 plots, with EUNIS level 2 habitat information included as a predictor. The number of plots for each EUNIS level 2 habitat is shown in each panel.

Of the five environmental variables assessed as CM_EIV_s over the past six decades (1960–2020), nitrogen showed the most substantial shift (Fig. 1). Specifically, we detected a substantial increase in CM_EIV_ for nitrogen across plots (mean change: +0.25 CM_EIV_; Fig. 1A), with 62% of plots increasing by at least 0.1. This trend was consistent across all four habitat types (Fig. 1B) and across Europe (Fig. 2), despite some variation in the magnitude of change in specific habitat types (Fig. 3). Increases in nitrogen values were particularly pronounced in deciduous forests, dry grasslands, and several other scrub and wetland types (Fig. 3). Only a few specific habitats showed either no substantial change (mesic grasslands) or even decreases (wet grasslands, woodland fringes, and tall-forb stands) in nitrogen EIVs.

Although changes in other EIV variables were generally smaller in magnitude, we observed context-dependent trends that varied by habitat type and geographic region. Light availability generally declined over time, showing an opposite trend to nitrogen CM_EIV_, which suggests that vegetation has become denser and less suitable for light-demanding species. However, the magnitude of change in light was substantially smaller than the change in nitrogen across habitats (mean: −0.12 CM_EIV_). The negative trend for light was most pronounced in grasslands (Fig. 1B) and in wetland habitats for bogs and fens (Fig. 3). In contrast, predictions for forests showed no overall change (Fig. 1B), although coniferous forests exhibited a positive trend and broadleaved evergreen forests a negative one (Fig. 3). Temperature change over time showed low variation across the analyzed plots, with most exhibiting minimal change close to zero across all habitats (Fig. 1A). Nonetheless, a small but detectable increase in CM_EIV_ for temperature was observed in forests (Fig. 1B). In addition, CM_EIV_ for temperature clearly increased in some specific habitats, particularly in alpine and subalpine scrub and grasslands over the past two decades (Fig. 3). Moisture CM_EIV_ declined substantially across wetlands (mean 1960–2020 change: −0.20 CM_EIV_), with pronounced negative trends particularly in helophyte beds (−0.06 CM_EIV_ per decade; table S1). Conversely, moisture CM_EIV_ increased in some dry, nutrient-poor habitats, such as dry grasslands and Mediterranean scrub vegetation, but also in periodically exposed shores (Fig. 3). A generally negative, albeit weak, trend in CM_EIV_ for reaction was observed in grasslands and wetlands overall (Fig. 1B). In contrast, a positive trend was detected in forests and in specific habitats typically associated with more acidic conditions, including tundra, bogs, and fen scrub (Fig. 3). The predicted CM_EIV_ changes for finer habitat types [European Nature Information System (EUNIS) level 2 habitats] were linked to their baseline levels, except for the nitrogen indicator (fig. S1).

**Fig. 4.**
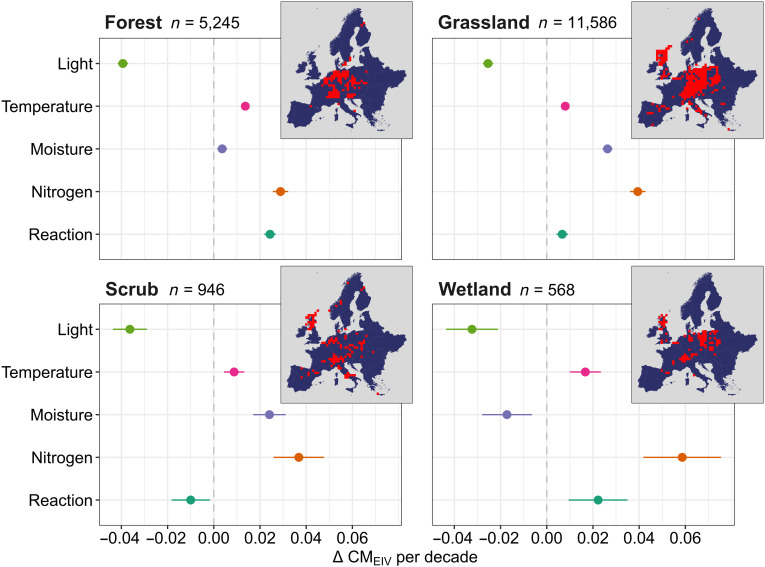
Trends in CM_EIV_s modeled across 18,345 resurvey plots collected in the field (ReSurveyEurope) using linear mixed-effect models. Observation records included in the model spanned the period from 1960 to 2020. The main panel shows the estimated slopes (and 95% CI; *x* axis) representing the change in CM_EIV_ every 10 years for each EIV variable (*y* axis) and for each main habitat type. The inset maps illustrate the total number and spatial distribution of ReSurveyEurope plot records available for each habitat in Europe in 75-km–by–75-km grid cells.

The results obtained from spatiotemporal interpolation were consistent with the overall direction of change of CM_EIV_s estimated from time series data available across Europe (Fig. 4). We found that CM_EIV_ for nitrogen consistently increased in all habitat types with time, accompanied by a consistent decrease in CM_EIV_ for light. Unlike our main interpolation analysis, CM_EIV_ for light in forests showed a substantial negative trend over time (−0.04 CM_EIV_ per decade), CM_EIV_ for moisture showed an overall tendency to increase in grasslands, and temperature showed more pronounced positive trends across habitats (Fig. 4).

## DISCUSSION

### Widespread nutrient enrichment across Europe

Our study represents the first continent-wide assessment of long-term temporal dynamics in environmental preferences of plants reflected in community composition changes and spanning multiple abiotic gradients simultaneously. Among the environmental conditions assessed through bioindication, an increase in nitrogen values has emerged as the strongest and most common trend across European plant communities over the past 60 years. This finding aligns with a substantial body of local and regional resurvey studies that have identified nutrient enrichment and, more broadly, vegetation density and biomass increase (*36*) as widespread drivers of community composition change across different vegetation types (*9*–*11*, *13*, *14*, *37*). We have now confirmed this trend on a continental European scale using an unprecedented amount of data.

The shift toward more nitrogen-demanding plant communities is likely driven, at least in part, by rising nitrogen inputs from cropland fertilization and airborne deposition, which have caused extensive accumulation of reactive nitrogen in European terrestrial ecosystems since the beginning of the 20th century (*38*, *39*). Increases in nitrogen levels are widely recognized as key drivers of shifts in competitive dynamics of plant communities, favoring generalist and nitrophilous species with efficient nutrient uptake capability over habitat specialists across a variety of habitats (*11*, *40*, *41*). Nonetheless, we also acknowledge that the nitrogen indicator value can also partly reflect other related nutrients, such as phosphorus and potassium (*42*, *43*). In addition, nutrient indicator values reflect increases in vegetation density (*43*, *44*) and may, therefore, be driven by factors beyond direct anthropogenic eutrophication, for example, increased atmospheric CO_2_ levels (*45*) and biomass accumulation caused by management cessation (*37*). Such a confounding effect was corroborated by a concomitant decrease in light conditions (particularly in resurvey plots; Fig. 4 and fig. S2), which reflects an increase in vegetation density and biomass accumulation associated with a decline of light-demanding species. Overall, the near-ubiquitous rise in nitrogen indicator values reflects the combined (and likely interacting) effects of drivers promoting higher herb- and shrub-layer biomass productivity and accumulation (*43*) and is largely consistent with widespread reductions in management intensity across European vegetation (*14*, *35*, *46*).

### Habitat-specific trends in ecological preferences

We found strong habitat-specific responses for both the magnitude and direction of EIV changes. This finding aligns with previous studies, highlighting the habitat-specific responses of European vegetation to temporal environmental changes (*6*, *10*, *35*). While the observed directions of change generally aligned with our expectations (Table 1), habitat-specific trends suggest that some communities may be shifting toward more intermediate indicator values (fig. S1), although this pattern is not consistent across all habitats. For instance, moisture values declined significantly in wetlands (typically harboring species with higher moisture requirements than other habitats), suggesting the impact of hydrological modifications induced by climate and land-use changes (*12*). Conversely, moisture increased more in drier and less productive habitats, including Mediterranean scrub and dry grasslands, reflecting an increased prevalence of generalist species adapted to more mesic conditions (*10*, *47*, *48*). Likewise, nitrogen EIVs increased most strongly in nutrient-poor habitats, reflecting their higher vulnerability to eutrophication (*35*, *40*, *41*), whereas the few fertile habitats showed little or negative changes, as found for woodland clearings and tall forb stands (Fig. 3).

Such contrasting tendencies could partly reflect high species turnover, including the loss of habitat specialists and gains in generalist species affecting various European vegetation types (*4*, *10*, *11*). Specifically, an increase in generalist species in the vegetation could shift the CM_EIV_ values toward more intermediate conditions, particularly in habitat types characterized by more extreme conditions (e.g., moisture in wettest or driest habitats, or light in open versus closed-canopy vegetation; Fig. 3). Habitat-dependent trends could also reflect differences in plant strategies and life histories: For example, short-lived pioneer species with higher nitrogen-use efficiency can respond rapidly to environmental change, whereas communities dominated by long-lived perennials may exhibit slower or more constrained CM_EIV_ responses (although removing tree and shrub species did not affect our results in forests and scrub habitats; see fig. S3). From this perspective, differences across habitat types could reflect both ecological change (e.g., habitat degradation) and life-history dependent dynamics of species assemblages along environmental gradients.

The habitat classification system used (*49*) was developed independently of the EIVs. This independence is crucial for avoiding the influence of the “regression to the mean” (RTM) effect (*50*), where the response variable artificially shifts toward the mean if subgroups are selected based on extreme values and the time series is limited to only two snapshots (e.g., historical versus modern surveys). In contrast, our analysis relied on long-term time series involving multiple surveys that span the full gradient of environmental conditions across Europe. Furthermore, baseline community-mean indicator values were estimated by interpolating across all plots within a habitat at a given time, rather than relying on individual resurvey comparisons that could be affected by random fluctuations causing RTM (*50*).

### A weak support for a warming trend?

Contrary to our expectations, we did not detect evidence of a temperature increase across most plots and vegetation types based on EIVs. Compared to other putative drivers, our results could suggest that global warming has played a smaller overall role in changes in community indices across all habitat types in Europe so far. This result is consistent with resurvey studies comprising diverse habitats that often found nonsignificant or even negative trends in bioindicated temperature values (*14*, *37*, *48*, *51*). However, it is likely that global warming could have had indirect effects, mainly by favoring plant biomass buildup and causing increased vegetation density, particularly when coinciding with higher nutrient and CO_2_ fertilization effects (*45*). Furthermore, we cannot definitively exclude a potential direct warming effect, since a marginal increment in temperature values was found in both the interpolation analysis (Fig. 1) and in the time series data (Fig. 4). Unlike nitrogen-related changes, which can trigger rapid species turnover due to the immediate availability of colonizer pools within local habitat mosaics, the response of biological communities to global warming may exhibit lagged and slower dynamics. This is the case for lowlands (*52*), as temperature gradients in flat terrains often extend over large spatial extents, thus limiting the immediate availability of thermophilous colonizing species nearby and consequently reducing the detectability of warming through bioindication. Conversely, in mountain environments, elevational gradients facilitate more readily observable upward shifts caused by global warming, especially for thermophilous and nutrient-demanding species typically found at lower elevations (*53*). Our study consistently detected clear warming trends specifically in alpine scrub and grasslands, particularly over the past two decades.

### Limitations of EIV-based causal inference

It is important to acknowledge that EIV variables at the species level are partially correlated (fig. S4). At the community level, EIVs exhibited even stronger correlations depending on habitat type (fig. S5). This inherent multicollinearity complicates the straightforward attribution of observed community-mean shifts to distinct putative drivers. Because environmental drivers often act simultaneously, changes over time in one EIV (e.g., nitrogen) are frequently accompanied by correlated shifts in other EIVs (e.g., light or moisture; fig. S2), making it difficult to isolate individual effects. We therefore caution against uncritically drawing direct causal links based solely on EIV variables. A clear example of this challenge can be seen in wetlands: Community changes indicated by shifts in nitrogen were found to be positively correlated with both soil reaction and temperature. This implies that an observed change in a wetland community might be simultaneously signaling a change in soil pH, a warming effect, or a complex combination of both, rather than solely indicating eutrophication. Thus, while the dynamics of different variables serve as proxy indicators and enable the comparison of their potential importance, we emphasize that the interpretation of EIV variables and their observed changes must be made with caution. This further underscores the need for European monitoring programs to assess local site conditions directly, in addition to biodiversity observation.

Furthermore, we acknowledge that the ecological optima of some species could vary across regions (*54*) and over long time scales and that such intraspecific variability and potential time-lag responses are not captured by static EIV assignments. However, we expect this limitation to have little influence on our results, because the trends analyzed here primarily reflect changes in species composition at the community level over a few decades. Moreover, any within-species variation in EIVs is averaged across multiple species within each plot, making intraspecific adjustments negligible in the context of our large-scale synthesis.

### Environmental implications

Our study provides strong evidence that European plant communities have broadly shifted toward more nitrogen-demanding species composition over the past 60 years. The widespread increase in the prevalence of such species is likely the outcome of several global change drivers that may interact in complex ways, with the specific drivers often varying by context. Furthermore, the trends in light and moisture indicators reinforce the conclusion that land-use change has significantly shaped plant community composition, presumably contributing to habitat deterioration and loss of habitat specialists across various habitat types. Thus, alongside the often-studied effects of global warming (*55*, *56*), eutrophication and land-use management changes should be accounted for when addressing the impacts of global environmental changes on large-scale dynamics of biological communities.

From a practical standpoint, landscape-planning interventions, such as establishing ecological buffer zones (e.g., hedgerows and road verges) around protected areas in agricultural landscapes, could shield them from nutrient runoff from agricultural fields (*57*). Nonetheless, reducing nitrogen surpluses at their source, particularly from intensive agriculture and fossil fuel combustion, remains essential to reduce long-range nitrogen deposition and safeguard plant biodiversity across European ecosystems (*38*). Furthermore, we underscore the need for local conservation and ecological restoration strategies that mitigate soil eutrophication, maintain open vegetation, and preserve characteristic moisture regimes (moist in wet ecosystems and dry in arid ones). These could involve reintroducing or maintaining intermediate disturbance regimes via traditional management practices, such as coppicing (*58*, *59*), litter removal (*60*), and extensive mowing or grazing (*61*), or less commonly used management tools for Europe, including prescribed burning (*62*) and trophic rewilding (*63*). Coordinated local and policy-level actions are therefore necessary to effectively shape long-term trends in plant community composition change.

## MATERIALS AND METHODS

### Vegetation plot data

We based our analyses on records of vascular plant species from an initial set of 1,679,403 vegetation plots stored in the EVA (*29*) and ReSurveyEurope (*28*) (project no. 222, version 2024-09-19; https://doi.org/10.58060/hgrb-sw46). We selected plots with complete information on geographical location, habitat type, plot size, and sampling year, applying specific filters based on each of these variables, as described below. These criteria resulted in a subset of 622,906 vegetation plots in EVA sampled once and 69,487 observations from 21,618 plots in ReSurveyEurope each sampled at least twice. A version including observations from both datasets without temporal replication was used for model training and interpolation. In addition, we used a subset of the ReSurveyEurope dataset to test whether our interpolation approach could predict actual EIV changes within time series data (see “Random forests validation”).

1) Geographic location. We included only plots located in Europe (excluding Iceland, Macaronesia, Svalbard, Russia, and Turkey) with available geographical coordinates (fig. S6). In addition, we excluded plots with a geographic uncertainty greater than 1 km. We retained plots without reported uncertainty, assuming that, in most cases, their true positional error is below this threshold. The coordinates of repeated observations for the same plot in ReSurveyEurope are not always consistent, which can be attributed to relocation errors. Thus, to minimize potential noise in EIV interpolation during model validation, we excluded any plots from ReSurveyEurope where at least one of its survey observations was reported to deviate by more than 100 m from other associated observations for that same plot.

2) Habitat types. Vegetation plots were classified into habitat types using the expert system for the EUNIS Habitat Classification (EUNIS-ESy; version 2021-06-01) (*49*), which mostly relies on species composition and cover. We focused exclusively on plots categorized as forest, grassland, scrub, and wetland at level 1 of the EUNIS classification. Coastal dune/sandy shore habitats (i.e., “N1”) were reclassified, where possible, into these types based on their physiognomy (e.g., coastal forests were classified as forest rather than as coastal habitats). Plots from the Danish Nature Database lacking species cover for EUNIS classification were assigned to habitats using the European Union (EU) Habitats Directive’s Annex I habitat conversion (*64*). In the final selection, the dataset included 181,881 forest, 319,886 grassland, 67,645 scrub, and 122,981 wetland plots.

3) Plot size. We excluded all plots with no information on plot size. We included only plots with sizes ranging from 100 to 1000 m^2^ for forests and from 1 to 100 m^2^ for grasslands, scrub, and wetlands, to exclude outliers and potential reporting errors regarding plot size in EVA and ReSurveyEurope. These plot-size ranges are standard in European vegetation surveys (*65*) and were explicitly accounted for in the analyses to minimize potential sampling bias.

4) Sampling year. We only included plots sampled between 1945 and 2023. Plots sampled prior to 1945 were excluded due to their much lower number compared to subsequent periods. Although EIV changes were analyzed from 1960 to 2020, the model was calibrated using data from 1945 to 2023. This wider range, analogous to training models over larger geographical extents, potentially enables the model to more robustly capture underlying long-term temporal dynamics by mitigating edge effects at the boundaries of the studied period (1960–2020). The temporal distribution of the included ReSurveyEurope data broadly matched that of the EVA data (fig. S7), and we achieved comprehensive temporal and geographical coverage of plots across most European regions (figs. S8 and S9). For the selected time series within the ReSurveyEurope dataset, the median time span between the first and last observation was 16 years (ranging from 1 to 73 years; SD = 14.6 years).

5) Additional filters on time series data. For the ReSurveyEurope data, plots were excluded if, after applying the previously outlined criteria, all their remaining observations fell within the same year of sampling. The data included both permanent plots (15,856; 73% of these time series), resurveyed at precisely relocated sites, and quasipermanent plots (5762; 27%), which lacked accurate relocation information. All experimentally manipulated permanent plots were excluded from the ReSurveyEurope dataset.

We provide a summary of the numbers of plots and species used for each methodological step in table S2.

### Ecological indicator values

Data on EIVs were sourced from Ecological Indicator Values for Europe (EIVE; version 1.0) (*25*). This consensus system covers the European flora and was derived from 31 regional, expert-based EIV systems. It contains five ecological indicators, each on a continuous scale from 0 to 10, where 0 represents the lowest and 10 the highest value found on the continent. Because EIVs are on a decimal scale, they can be directly expressed as a percentage of the full European gradient; for instance, a change in mean EIV of 0.1 represents a 1% change of the entire European range for that variable. At least one EIV for light, temperature, soil moisture, soil nitrogen, and soil reaction was available for 13,874 of the 16,810 vascular plant taxa recorded in the selected vegetation plots. The 2936 taxa missing from EIVE were typically very rare in the vegetation plot records (they occurred in 4% of the total number of plots; frequency range: 1 to 1014 plots; mean = 18.2 plots; SD = 53.6 plots), and their exclusion thus had a minimal impact on the results (fig. S10).

We calculated unweighted CM_EIV_s across all plots for each indicator variable. To account for potentially lower bioindication accuracy due to missing EIVs, we included for each EIV variable only plots where EIVs were available for at least 80% of the species. This filtering led to the exclusion of only ~1% of plots per EIV variable. On average, the plots retained for analysis had EIVs for more than 98% of the vascular plant species co-occurring in the same plot. In the Supplementary Materials, we report pairwise correlations between EIVs both at the species (fig. S4) and community (plot) level (fig. S5).

We checked the consistency of our approach by comparing our main results (based on CM_EIV_) with those obtained using the community-weighted mean (CWM_EIV_), in which mean EIV values are weighted by species cover (abundance) within each plot. Cover values for the same species occurring in different vegetation layers were first merged using the formula proposed by Fischer (*66*). We then used a subset of 477,990 plots for which species cover data were available and repeated all statistical analyses described below on this subset for both CM_EIV_ and CWM_EIV_. Since the two approaches yielded very similar results (figs. S11 and S12), we used unweighted community means in the main analyses instead of cover-weighted means, following the principle that less abundant species also reflect environmental conditions (*22*) and that unweighted means offer more reliable bioindication (*67*, *68*). Last, because long-lived species (such as trees and shrubs) may locally persist after environmental changes, we recalculated CM_EIV_ after excluding 845 tree and shrub species (sourced from https://floraveg.eu/) and compared models obtained by excluding such species in forest and scrub vegetation (fig. S3). The results were again very similar, indicating that excluding the longest-living species in closed-canopy vegetation does not meaningfully influence our large-scale patterns.

### Random forests training and evaluation

We used random forests (*32*) to model the dependence of plot-level CM_EIV_s on space (i.e., elevation, latitude, and longitude) and time (i.e., sampling year) while accounting for the effect of plot size, species richness of vascular plants, and habitat type (either forest, grassland, scrub, or wetland). Tree-based machine learning algorithms, random forests in particular, are well suited to this task (*6*, *69*), as they can model complex interactions among predictors and capture nonlinear, grain-dependent effects more effectively than parametric methods. Prior to modeling, we transformed geographic coordinates to latitude and longitude in meters [using ETRS89/UTM zone 32N (EPSG:25832) projection, hereafter, “northing” and “easting,” respectively] and used this projection throughout the analysis. We extracted elevation at the plot location using a Digital Elevation Model with 90-m horizontal resolution from the European Space Agency (*70*). We accounted for the effect of vascular plant species richness and plot size (i.e., the species-area relationship) on the response variable (CM_EIV_), as species-poorer plots are more likely to experience larger changes in CM_EIV_. For each response variable, we trained our model on 644,524 vegetation plots: 622,906 from EVA and 21,618 from ReSurveyEurope, the latter obtained by selecting one plot observation at random from each time series. The remaining 47,869 plot observations from ReSurveyEurope were excluded from model training but were later used for independent model validation and time series analysis (see “Random forests validation” and “Mixed-effects model analysis on time series data” sections). We also fitted separate models on a subset of 384,254 plots, where EUNIS level 2 habitat information was available. In these models, we used EUNIS level 2 habitat as a predictor instead of broad habitat type. This allowed us to generate predictions that distinguish among finer habitat categories, for example, separating deciduous from evergreen forests or dry from mesic grasslands.

Analyses were conducted in R (version 4.4.2) (*71*) using the tidymodels R package collection (*72*), applying random forests via the ranger engine (*33*). The dataset was randomly split into training (80%) and test (20%) sets, stratified by CM_EIV_. Models were fitted using the following formula

$$\begin{array}{c} {\text{CM}}_{\text{EIV}}\;∼\;\text{northing}\;+\;\text{easting}\;+\;\text{elevation}\;+\;\text{sampling year}\\ \;+\;\text{species richness}\;+\;\text{plot size}\;+\;\text{habitat type} \end{array}$$

We included habitat type as a predictor to obtain habitat-specific estimates within a unified model, which improves feature learning in random forests and allows us to compare trends across habitats. We performed hyperparameter tuning with 10-fold cross-validation, testing 24 combinations of the number of randomly sampled predictors (i.e., “mtry” from 2 to 7) and node sizes (2, 5, 10, and 20) with 500 trees. We selected the best set of tuning parameters based on the lowest root mean square error (RMSE) and highest *R*^2^ [i.e., squared Pearson correlation coefficient of observed and predicted data; (*72*)] (fig. S13).

Model performance was evaluated using a 10-fold cross-validation with three repeats on the training data (table S3) and on the test set (final fit; fig. S14). The models predicted CM_EIV_ with high accuracy, with *R*^2^ ranging from 0.78 (RMSE = 0.62) for nitrogen and 0.93 (RMSE = 0.18) for temperature (table S3). We did not apply block cross-validation (*73*), as it is not appropriate for interpolation models that use geographic coordinates and time as predictors. In our case, blocking would artificially constrain the model’s ability to learn from continuous spatial and temporal gradients (*6*). We found no clear spatial pattern in model residuals obtained from the testing data (fig. S15), indicating that the model adequately accounted for spatial patterns in the underlying data and that its predictive accuracy was not systematically biased in any particular region. We assessed variable importance using the node impurity measures from the random forest model (fig. S16).

### Random forests validation

To validate our interpolation approach, we assessed its ability to predict changes over time using ReSurveyEurope data, which comprise actual vegetation survey time series collected in the field (*6*). For each EIV variable, we repeated the model training and testing step 100 times, exclusively on the ReSurveyEurope data. In each iteration, we randomly selected a single observation for each of the 21,618 time series, ensuring that models were trained on data without temporal replication, as in our main analysis (see the “Random forests training and evaluation” section). Model performance was then evaluated on (i) a 20% hold-out test set and (ii) the observed absolute change of CM_EIV_ between the initial and final survey in each time series. A positive correlation in (ii) indicates that CM_EIV_ changes predicted from models trained on data lacking temporal replication reflect actual CM_EIV_ shifts observed across time series data. We obtained good model accuracy, with Pearson correlations between observed and predicted values ranging from 0.51 (*R*^2^ = 0.26) for nitrogen and temperature to 0.61 (*R*^2^ = 0.37) for moisture (table S4 and fig. S17), indicating that predictions derived from static data capture overall changes in CM_EIV_.

### Interpolating random forests to predict EIV changes

We used the random forests models to predict CM_EIV_ trends from 1960 to 2020 across 610,537 vegetation plots sampled during that period, keeping all predictors at their original values except for plot size. To account for variability in plot size across habitat types, we set plot size to a fixed representative value for each habitat type when predicting EIVs in space and time: forests (225 m^2^), grasslands (16 m^2^), scrub (50 m^2^), and wetlands (25 m^2^). Because plot size in EVA and ReSurveyEurope can vary due to differing sampling protocols across contributing databases, the most representative plot sizes mentioned above were determined by calculating the weighted median of the mean plot size from each database, using the number of plots per database as weight. We quantified changes in CM_EIV_s from 1960 to 2020 by calculating the difference: ΔCM_EIV_ = CM_EIV2020_ − CM_EIV1960_ (Figs. 1A and 2). We also predicted CM_EIV_s for each plot and each year across the entire 1960–2020 period, allowing us to summarize continuous temporal trends using partial dependence plots (Figs. 1B and 3), rather than comparing only the endpoints (1960 and 2020). To visualize the geographic patterns of these changes across Europe (Fig. 2), we averaged plot-level ΔCM_EIV_ into 50-km–by–50-km grid cells, incorporating data from all plots and main habitat types within each cell. We also developed a web application in shiny (*74*) to interactively explore geographic patterns of ΔCM_EIV_ across different habitats and time periods at finer resolution (available at https://gmidolo.shinyapps.io/interpolated_EIV_change_app/).

### Mixed-effects model analysis on time series data

To compare conclusions derived from our interpolation approach to those directly computed from actual time series data collected in the field, we also performed a separate analysis of EIV trends exclusively on the ReSurveyEurope data. In this analysis, we used the entire set of 57,255 selected observation records across the 18,345 plots from ReSurveyEurope spanning from 1960 to 2020. We selected all plots that were surveyed at least twice and included all available observations from their time series, rather than limiting comparisons to the initial and final surveys. We restricted the analysis to observations from the same time series that did not experience changes in habitat type (e.g., transitioning from grassland to scrub). This was done to control for plots undergoing rapid changes caused by local land use conversions and plots specifically established to study the successional development of vegetation. We fitted linear mixed-effects models with an identity link function and Gaussian error structure using the lme4 R package (*75*). For each EIV variable, we modeled the CM_EIV_ using the following lme4 formula

$$\begin{array}{c} {\text{CM}}_{\text{EIV}}∼\text{habitat type}\;\times \;\text{sampling year}\;+\;{\text{log}}_{10}\left(\text{plot size}\right)\\ +\left(1|\text{dataset}/\text{plot}\right) \end{array}$$

We included an interaction term between habitat type and time (sampling year) to estimate habitat-specific CM_EIV_ change over time. We also accounted for the effect of (log-transformed) plot size (in square meters) and the nested random structure of the plot ID within each dataset ID composing ReSurveyEurope. Before fitting the models, we divided the sampling year by 10 so that the model coefficients represent decadal changes in CM_EIV_.
