# Supplementary material for: Sixty years of plant community change in Europe indicate a shift toward nutrient-richer and denser vegetation
Source: Sci Adv. 2026 Apr 10;12(15):eaeb2493. doi: 10.1126/sciadv.aeb2493 (PMC13068046; doi:10.1126/sciadv.aeb2493)
Supplement: Supplementary file 1 — Figs. S1 to S17 Tables S1 to S4 [file sciadv.aeb2493_sm.pdf]

Science Advances
AAAS

Supplementary Materials for

Sixty years of plant community change in Europe indicate a shift toward nutrient-richer and denser vegetation

Gabriele Midolo *et al.*

Corresponding author: Gabriele Midolo, midolo@fzp.czu.cz

Sci. Adv. **12**, eaeb2493 (2026)
DOI: 10.1126/sciadv.aeb2493

This PDF file includes:

Figs. S1 to S17
Tables S1 to S4

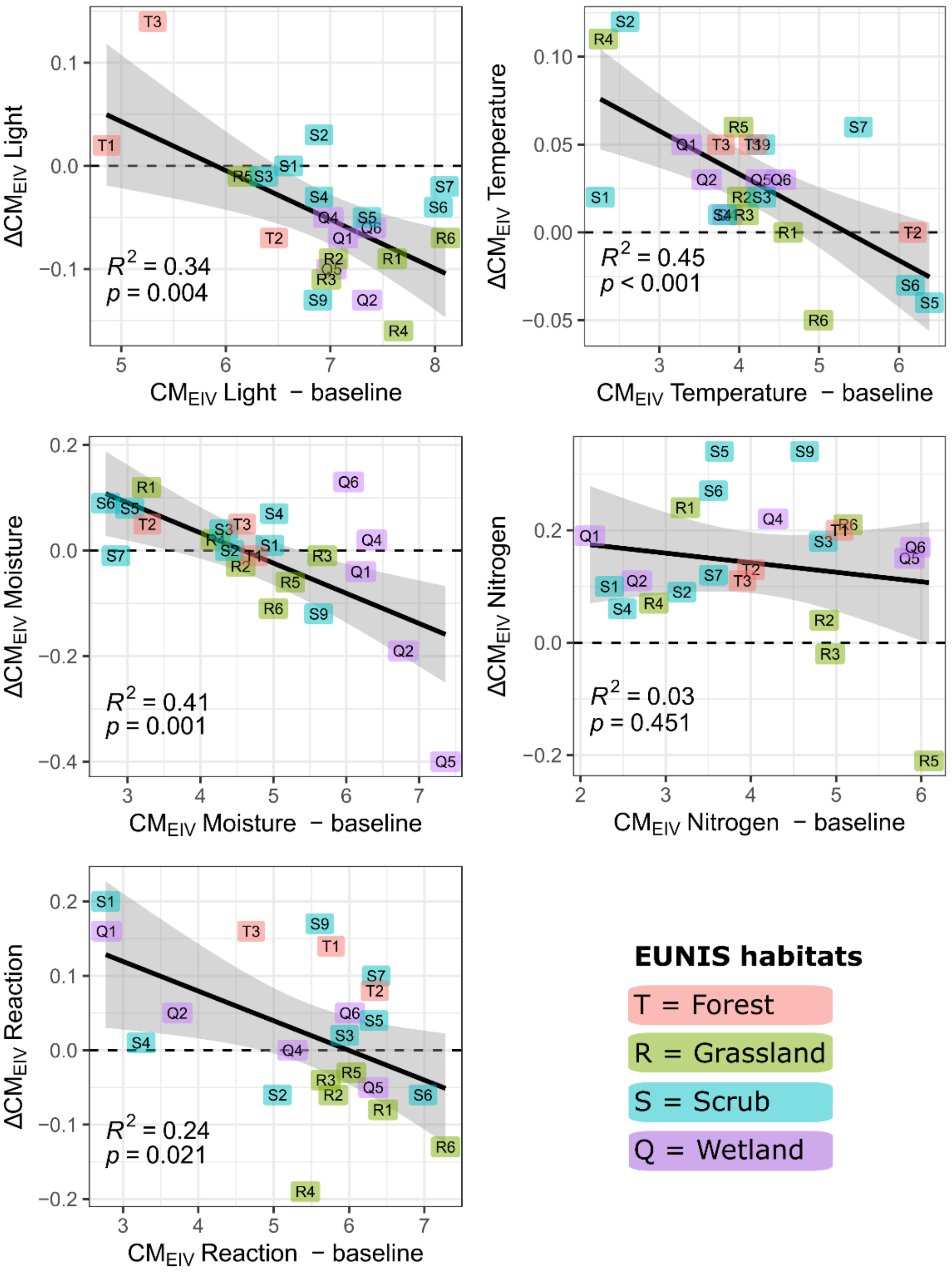

Fig. S1: Baseline CM_{EIV} versus predicted CM_{EIV} change across EUNIS (Level 2) habitats. The x-axis represents the mean CM_{EIV} across all plots belonging to each habitat predicted using Random Forests for the baseline year. The y-axis represents the mean predicted change in CM_{EIV}. The specific interpolation periods considered for each habitat, including baseline years, varied slightly (refer to Table S1). The overlaid regression line shows that, for all factors except nitrogen, habitat-level CM_{EIV} changes are linked to their baseline levels. See Figure 3 or Table S1 for the habitat codes.

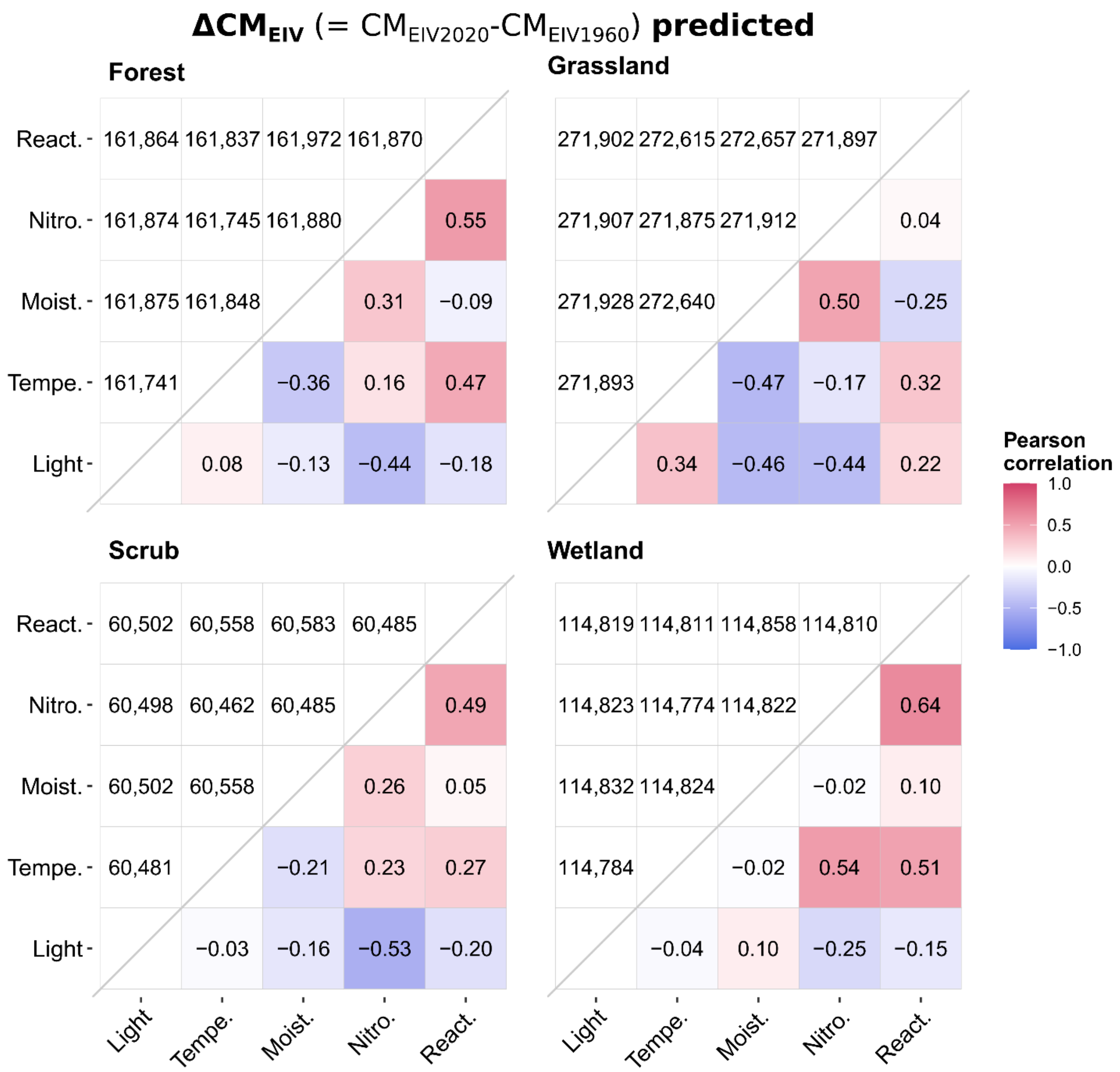

Fig. S2: Pairwise correlations of community-level indicator value changes (ΔCM_{EIV}) between 1960 and 2020. The changes are based on Random Forests predictions across different habitat types, utilizing a total of 610,537 vegetation plot records from the European Vegetation Archive (EVA) and ReSurveyEurope databases. The upper-left panel reports the number of plots with sufficiently complete information (available EIV values for at least 80% of the taxa present in the plot, and plot sampled between 1960 and 2020) to calculate the correlation coefficients.

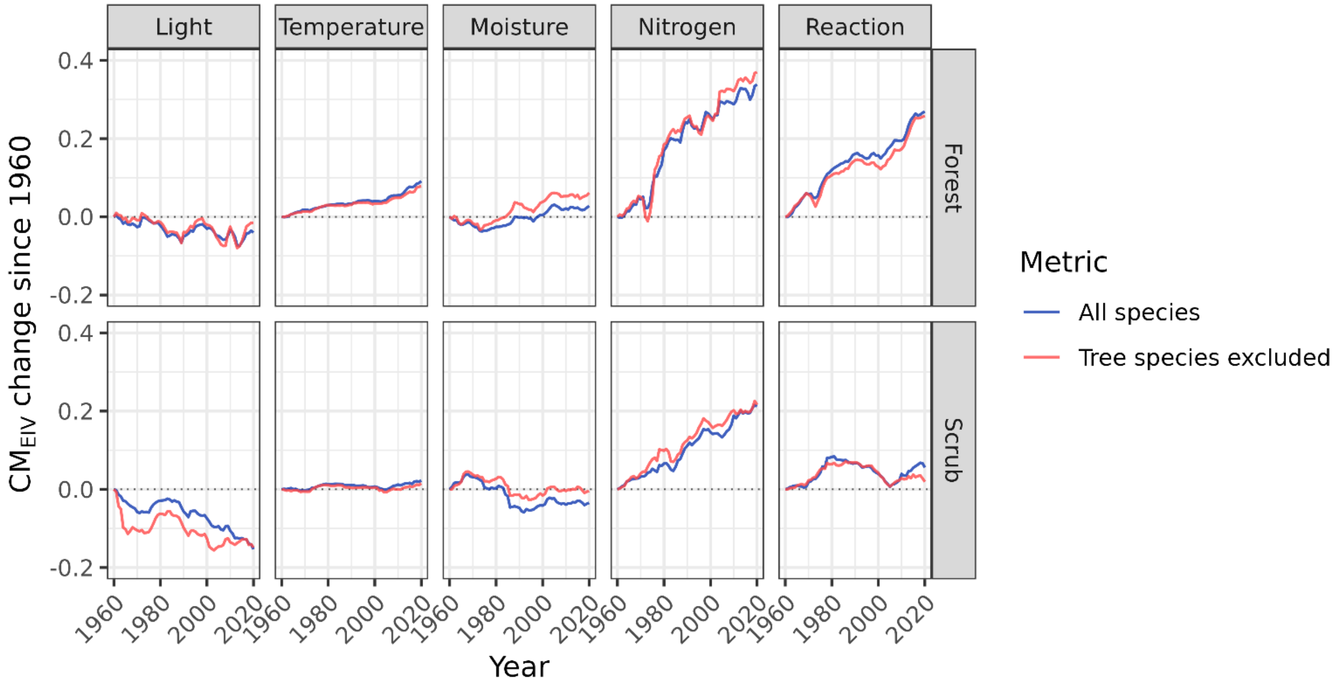

Fig. S3: Comparison of temporal CM_{EIV} trends between models including and excluding woody species. Interpolated temporal trends of community-mean ecological indicator values (CM_{EIV}) across 161,153 forest and 60,557 scrub vegetation plots sampled between 1960 and 2020 derived from two sets of Random Forests models trained by either including all life forms (as used in the main analyses; blue line) or excluding 845 trees and shrub species prior to CM_{EIV} calculation (red line). Only averaged predictions (means) across plots per year and habitat are shown for improved clarity.

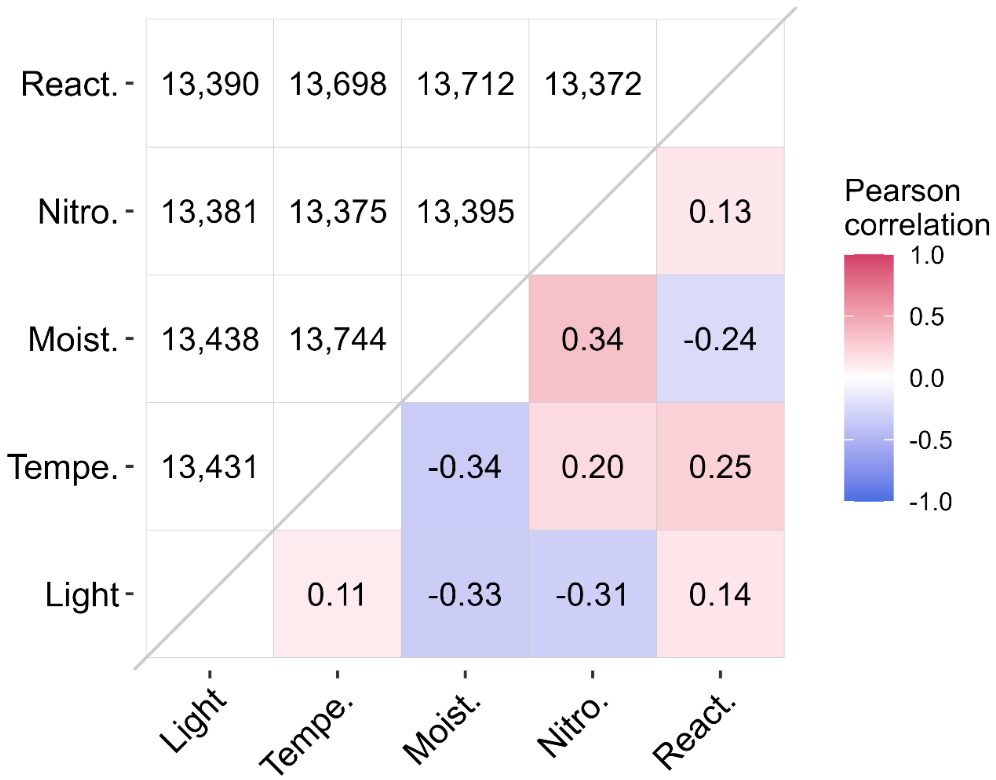

Fig. S4: Pairwise correlations of species-level ecological indicator values (EIVs) for 13,877 taxa. Pairwise correlation of species-level indicator values (EIVs) across 13,877 taxa used in the analyses and retrieved from the EIVE 1.0 database. The upper-left panel reports the number of taxa with complete information to calculate the correlation coefficients.

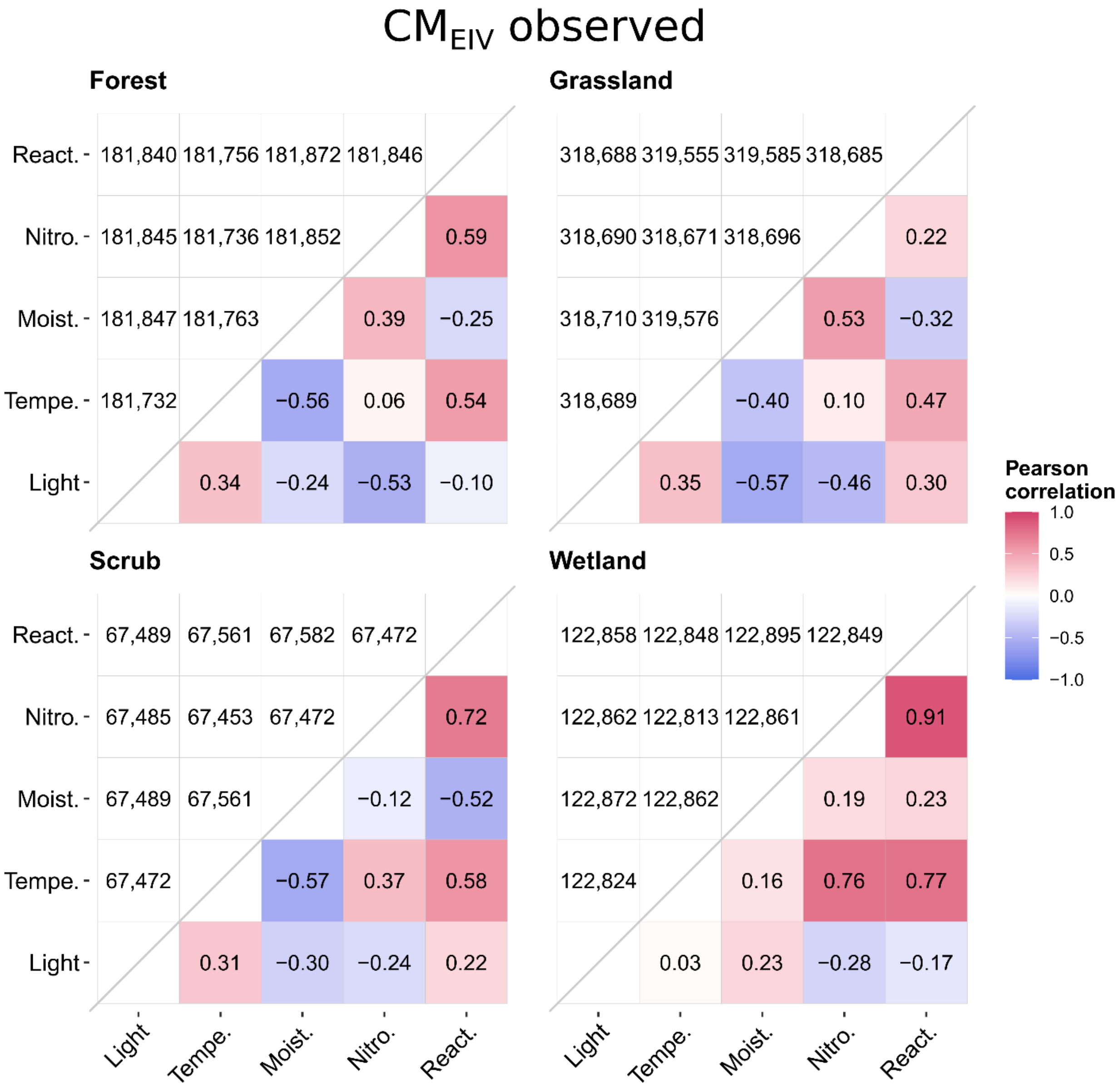

Fig. S5: Pairwise correlations of baseline CMEIVs across European vegetation plots. Pairwise correlation of community-level indicator values (CMEIVs) across different habitat types based on a total of 692,393 vegetation plot records from the European Vegetation Archive (EVA; no. plots = 622,906) and ReSurveyEurope (no. plots = 69,487) databases sampled between 1945 to 2023. The upper-left panel reports the number of plots with sufficiently complete information (available EIV values for at least 80% of the taxa present in the plot) to calculate the correlation coefficients.

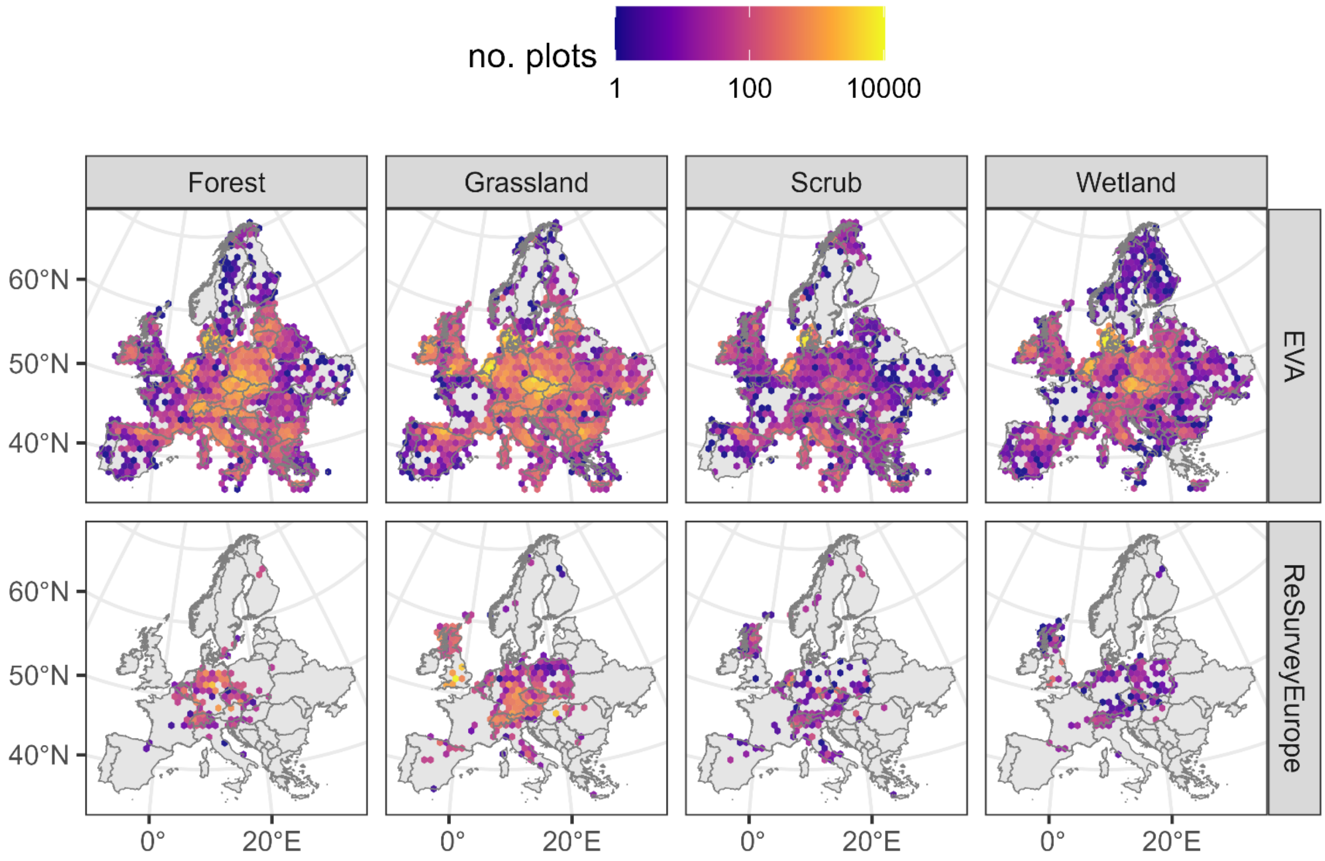

Fig. S6: Geographic distribution of vegetation plot records and habitat types. Geographic distribution of the plot records in the European Vegetation Archive (EVA) and ReSurveyEurope databases and habitat types used in the analyses, visualized using hexagonal binning.

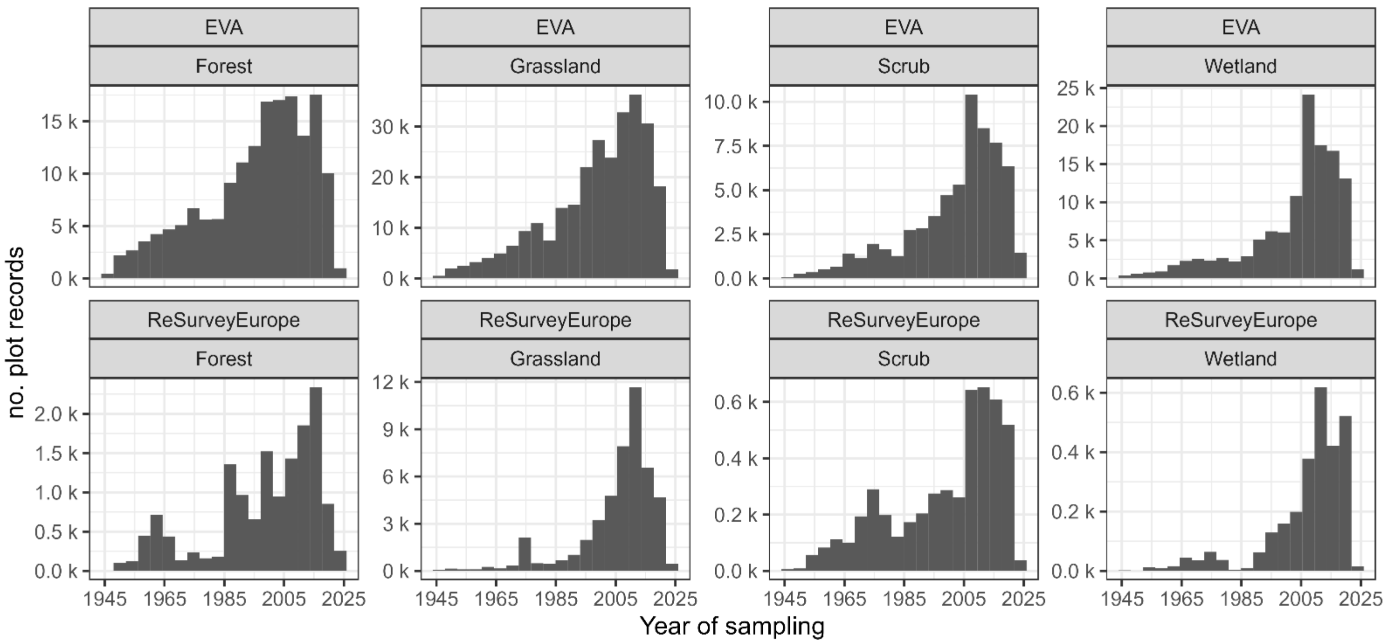

Fig. S7: Temporal distribution of vegetation plot records across databases and habitats. Temporal distribution of plot records across the European Vegetation Archive (EVA) and the ReSurveyEurope database and habitat types used in the analyses.

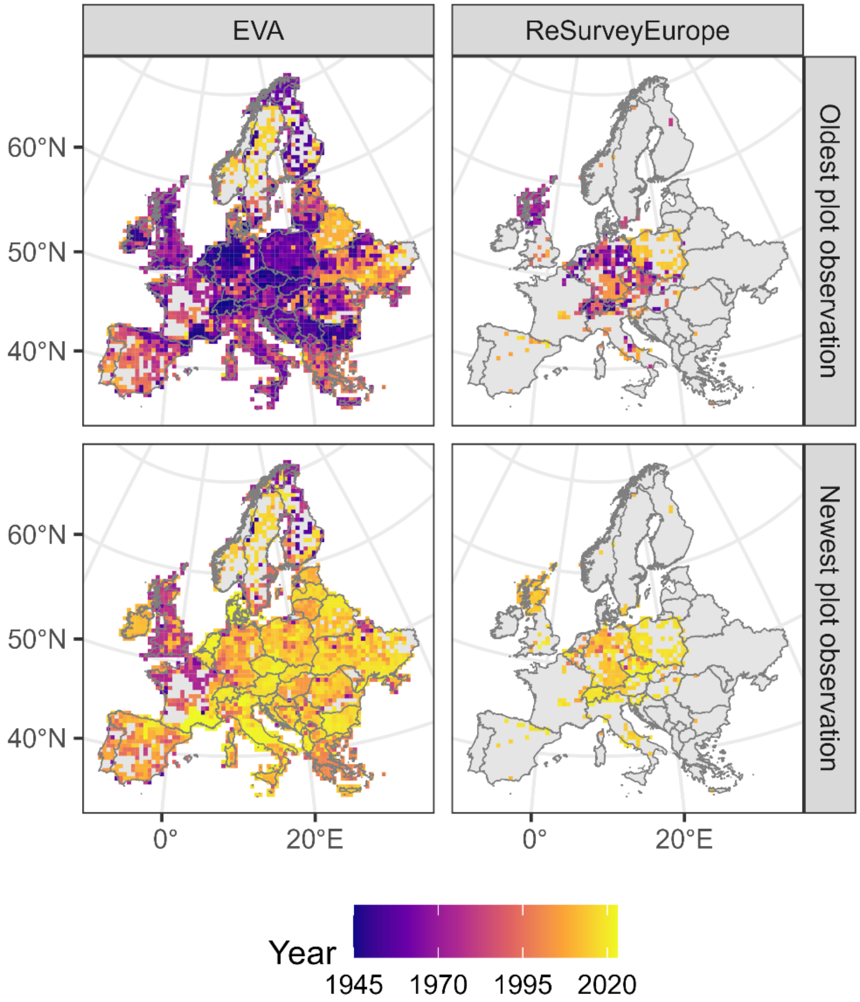

Fig. S8: Geographic distribution of the oldest and newest plot observations per grid cell. Year of the oldest (top panels) and newest (lowest panels) plot observation located in each grid cell (50 km × 50 km resolution) across the vegetation plots of the European Vegetation Archive (EVA) and the ReSurveyEurope database.

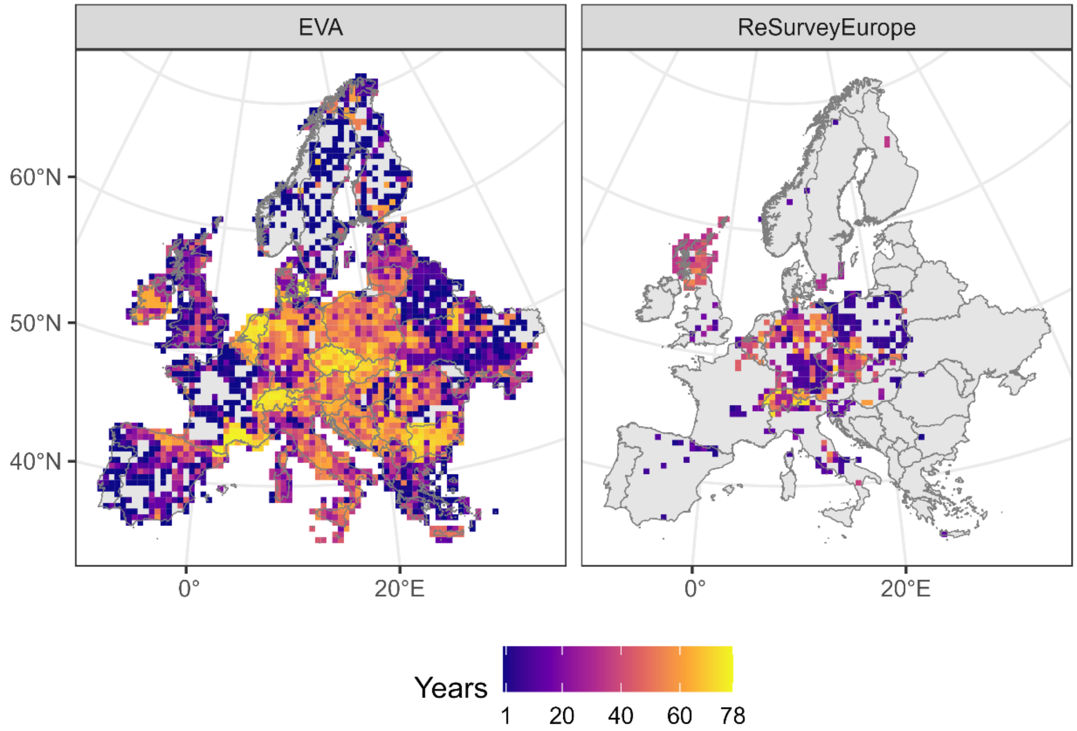

Fig. S9: Temporal span of plot observations across the study area. Temporal span (difference in years between the newest and the oldest plot observation) located in each cell on a 50 km × 50 km resolution grid across the vegetation plots of the European Vegetation Archive (EVA) and the ReSurveyEurope database.

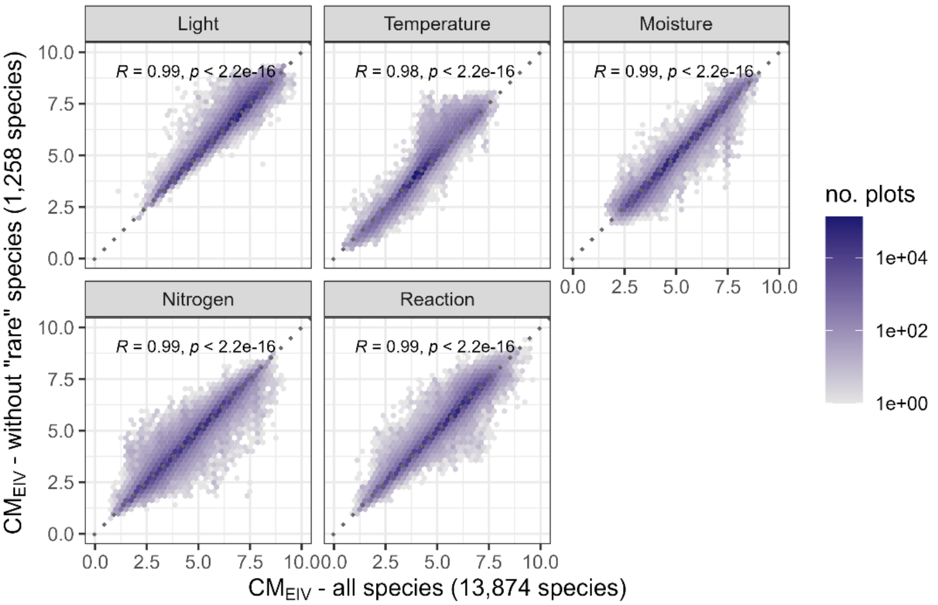

Fig. S10: Sensitivity of community-mean EIVs (CM_{EIV}s) to the exclusion of infrequent species. We removed all species recorded in fewer than 1,500 plots, retaining only 1,258 species and effectively excluding the vast majority (92.5%) of the regional species pool (compared to the 17.4% excluded in the main analysis due to missing EIVs). CM_{EIV}s derived from this reduced dataset (y-axis) were then compared with CM_{EIV}s calculated from the full species set used in the main analyses (x-axis). Despite the substantial reduction in species, CM_{EIV}s remained highly correlated (Pearson's $R \geq 0.98$), indicating that indicator values are largely determined by the relatively small subset of commonly available species that occur frequently across Europe.

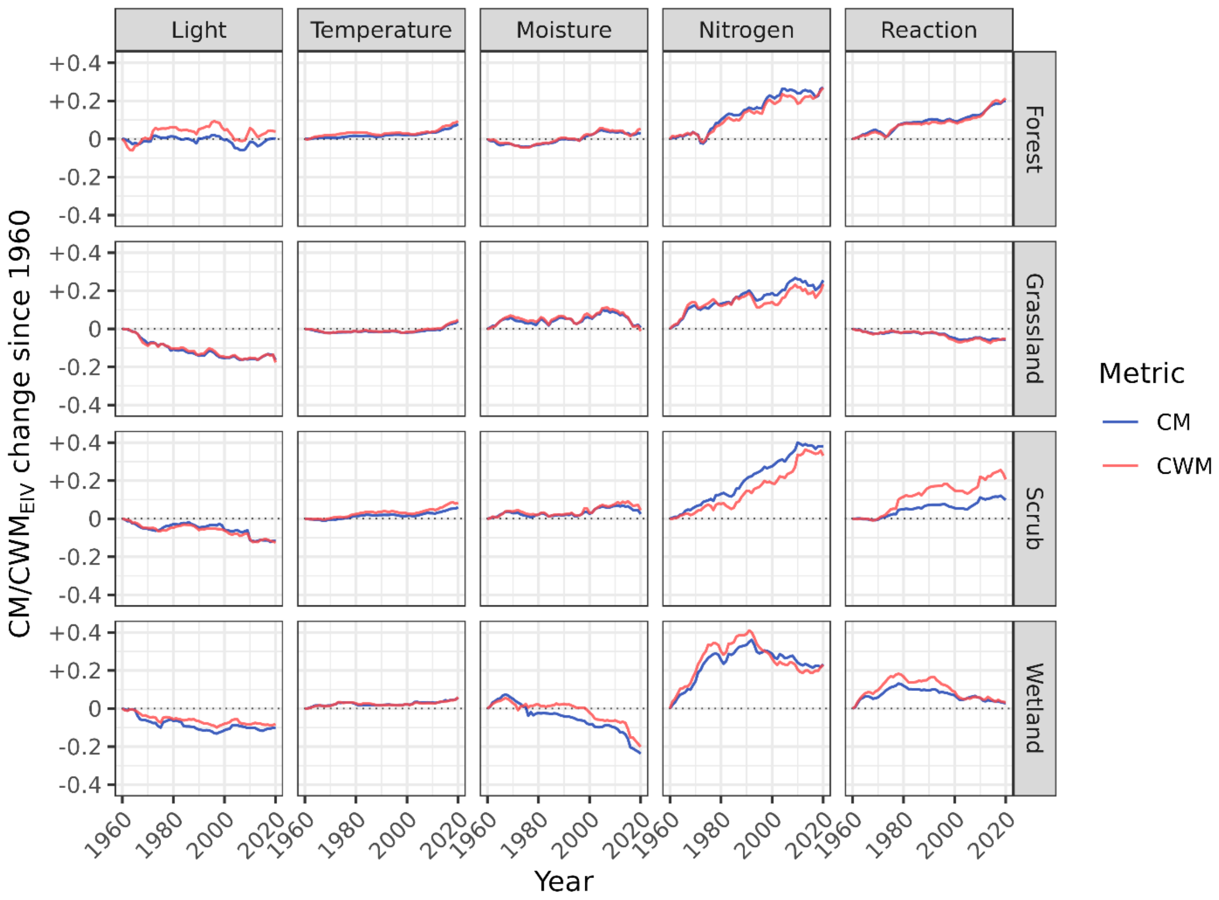

Fig. S11: Comparison of temporal trends between presence-only mean (CM_{EIV}) and cover-weighted mean (CWM_{EIV}). Interpolated temporal trends of community-mean ecological indicator values across 477,990 European vegetation plots sampled between 1960 and 2020 derived from two sets of Random Forests models trained on either community mean (presence only) (CM_{EIV}) or cover-weighted mean (CWM_{EIV}). Only averaged predictions (means) across plots per year and habitat are shown for improved clarity.

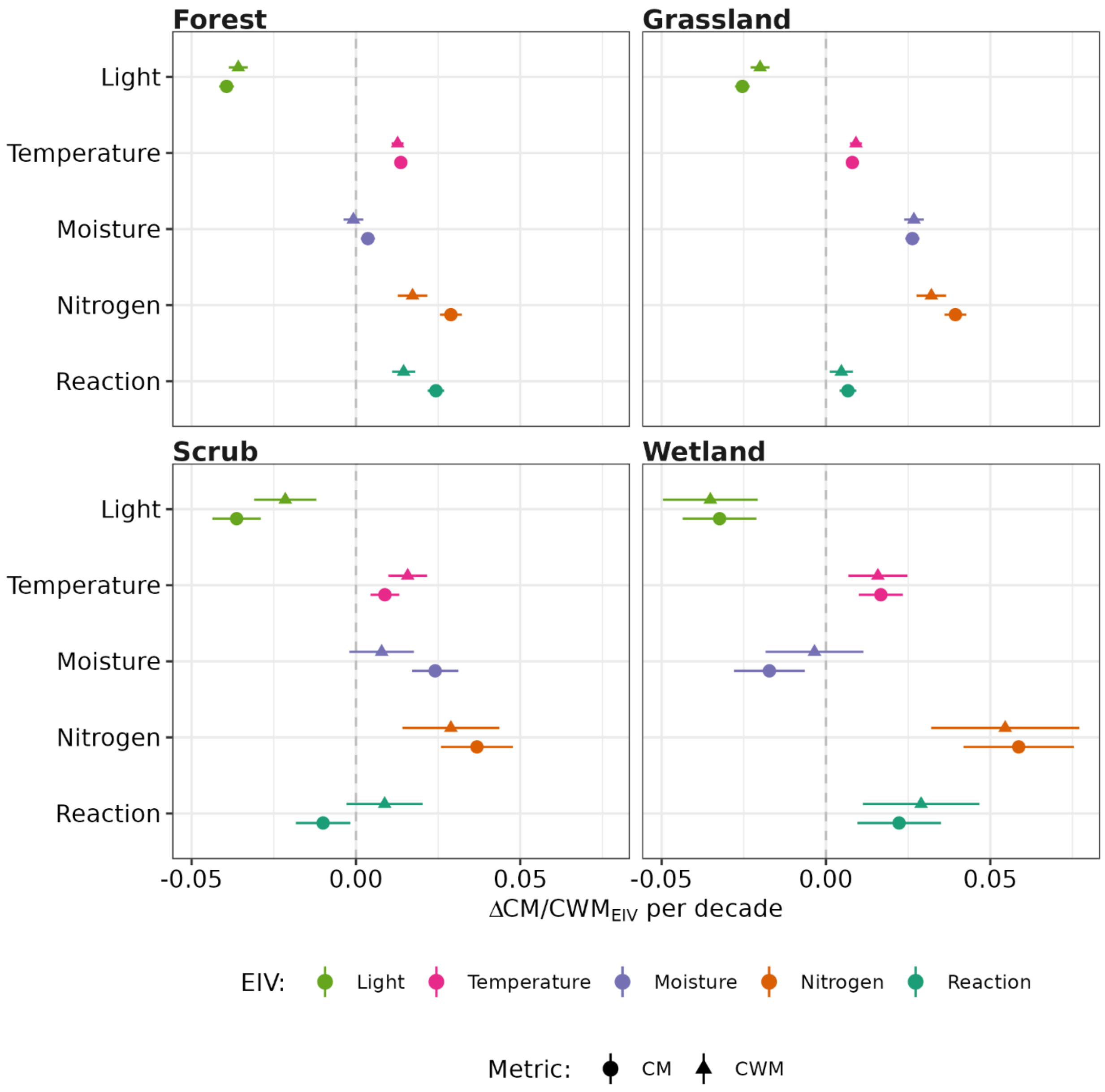

Fig. S12: Comparison of temporal trends between CM$_{EIV}$ and CWM$_{EIV}$ across resurvey plots. Trends in community-mean ecological indicator values (estimated slopes, and 95% CI), comparing presence-only community means (CM$_{EIV}$) (see also Figure 4 in the main text) and cover-weighted community means (CWM$_{EIV}$) are shown. Results are modeled across 18,345 resurvey plots (time series) data collected in the field and available in ReSurveyEurope, using linear mixed-effect models. Observation records included in the models spanned the period from 1960 to 2020.

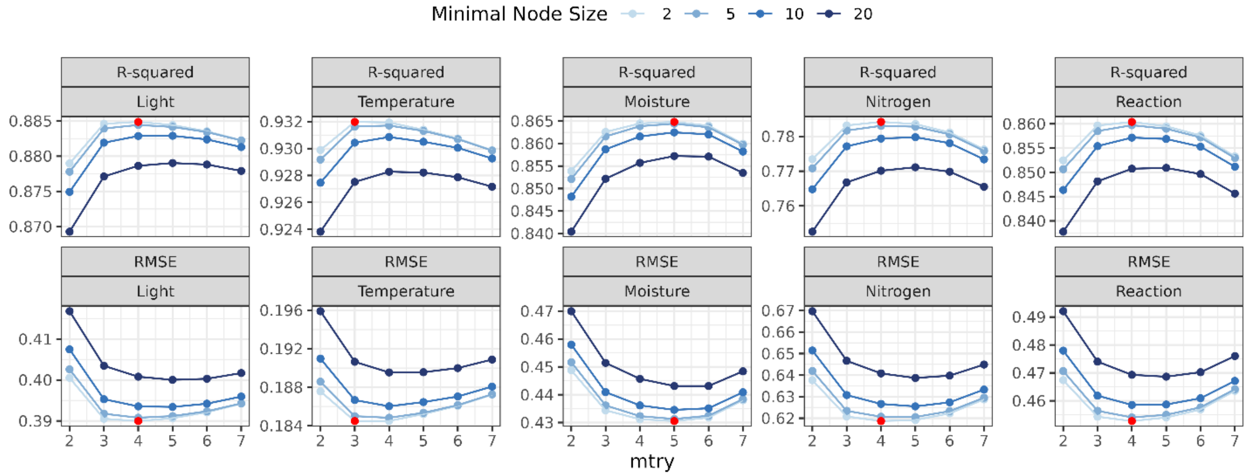

Fig. S13: Hyperparameter tuning results of minimal node size and the number of randomly selected predictors in Random Forests. The number of trees was not tuned and set to 500, as increasing this parameter substantially increased computation time with no improvement in RMSE. RMSE = root mean squared error; R-squared = squared Pearson correlation of observed vs. predicted values; $(\mathrm{cor}_{obs\ vs.\ predicted})^2$.

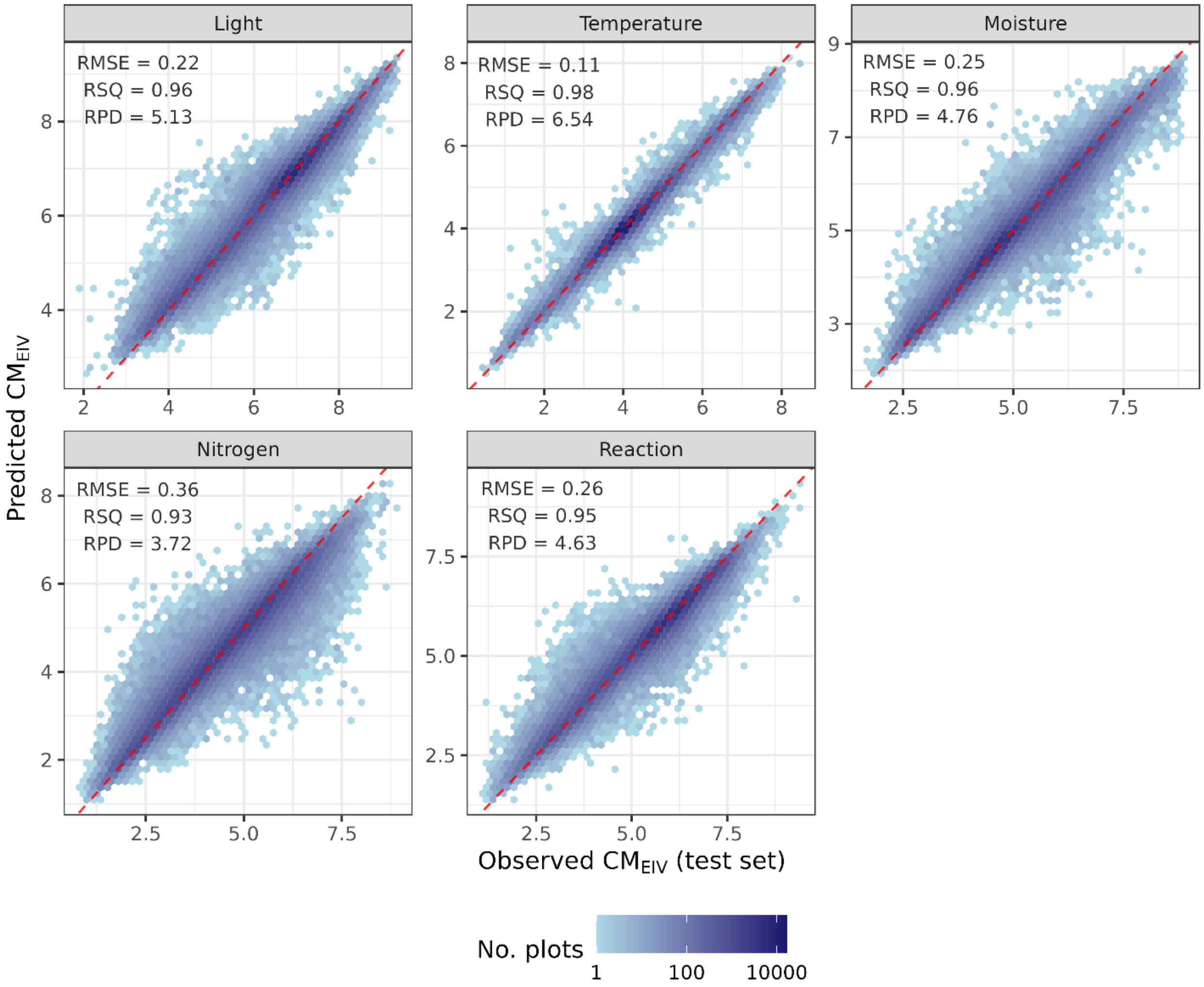

Fig. S14: Final fit on the entire training set evaluated on the test set. *RMSE* = root mean squared error; *RSQ* = squared Pearson correlation of observed vs. predicted values; $(\text{cor}_{\textit{obs vs. predicted}})^2$. *RPD* = ratio of performance to deviation (ratio of the standard deviation (SD) of the observed data to the standard error of prediction).

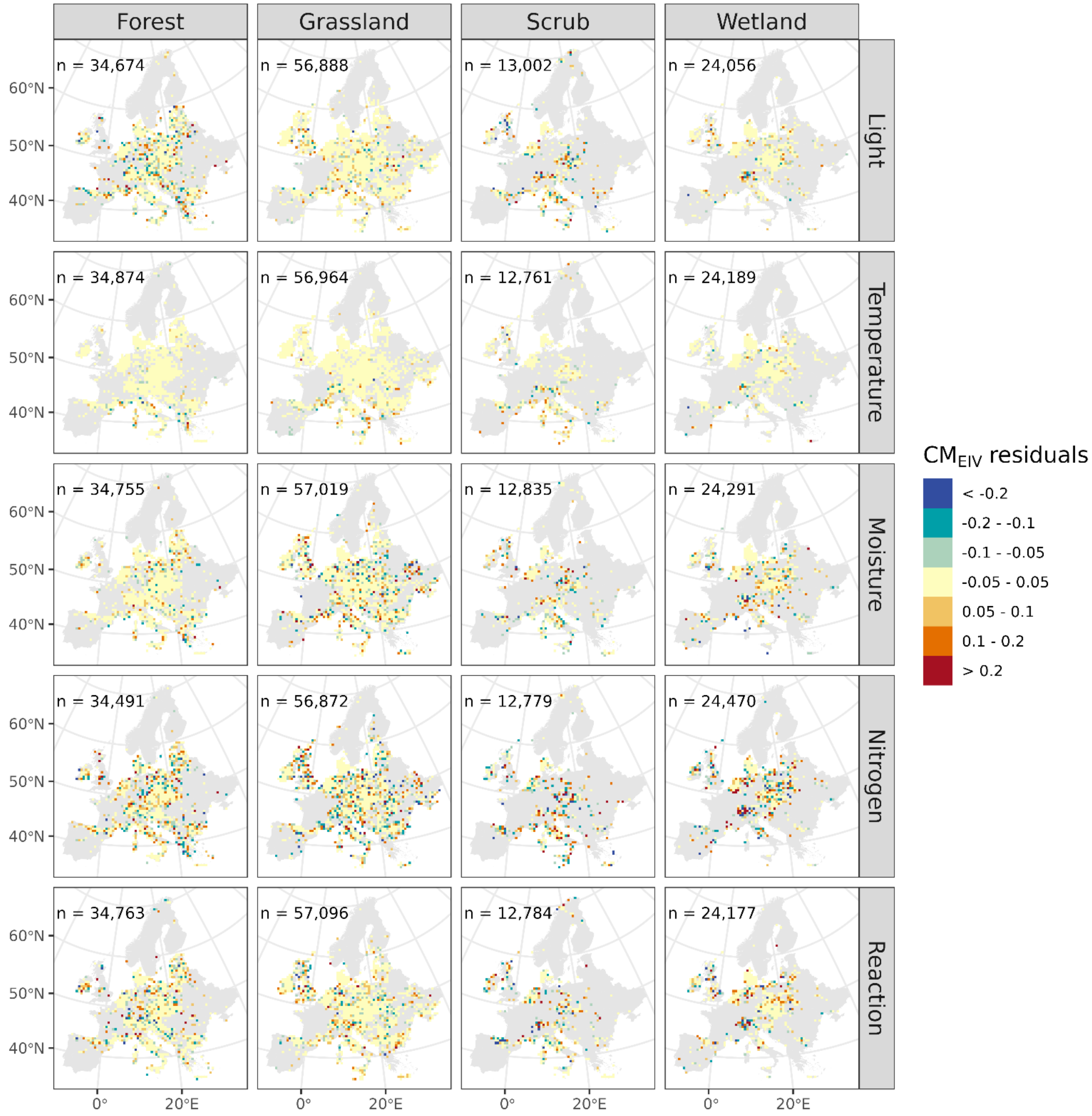

Fig. S15: Spatial distribution of Random Forest model residuals. The map shows the distribution of CM_{EIV} residuals (observed - predicted) across Europe from the Random Forest model estimated over the test data. Residuals were averaged within 50 km × 50 km grid cells for each habitat type. Only grid cells with at least five plots are included. The number of plots (*n*) is indicated within each panel. The lack of distinct geographic patterns in the residuals suggests that the model performed similarly in predicting CM_{EIV} across different regions.

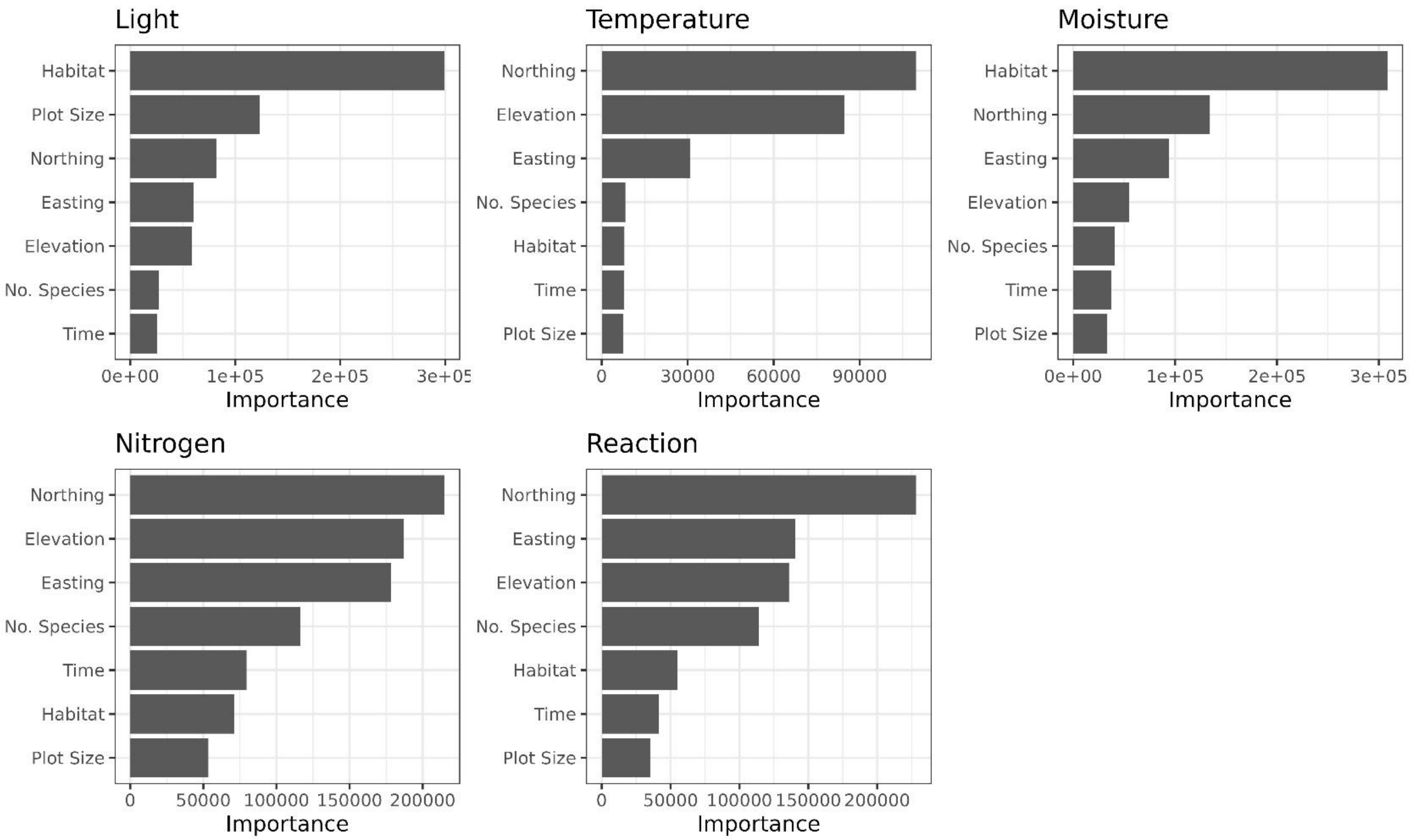

Fig. S16: Variable importance of predictors used in the Random Forest models. The figure reports variable importance (node impurity) for each indicator variable.

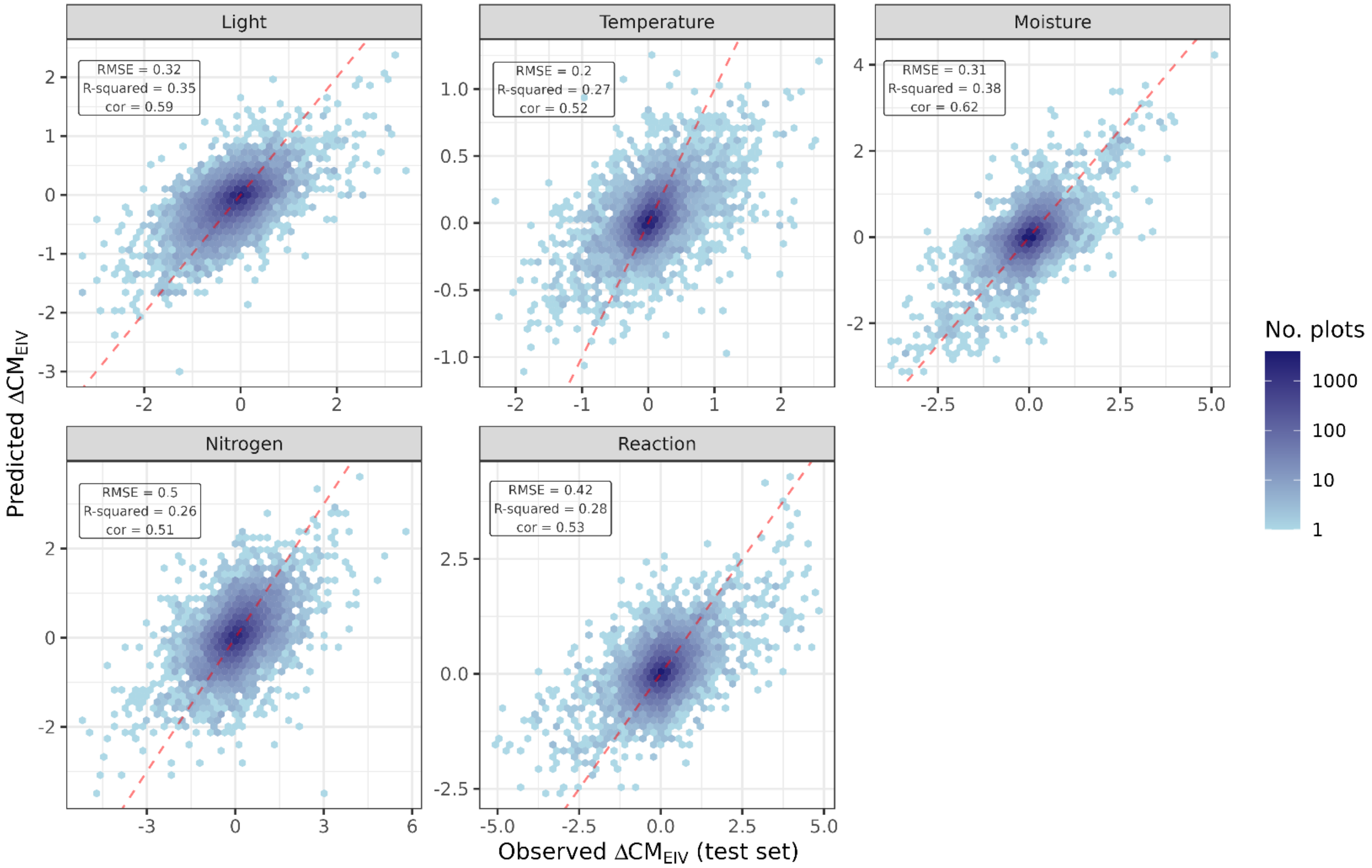

Fig. S17: Validation of CM$_{EIV}$ temporal interpolation using resurvey sites. Validation of CM$_{EIV}$ temporal interpolation tested across 21,618 resurvey sites available in the ReSurveyEurope dataset. The models are validated using the change in community-mean indicator values from the end to the start of each time series ($\Delta CM_{EIV} = CM_{EIV\,end} - CM_{EIV\,start}$). The figure and statistics display results obtained from a single iteration - see 'Model validation' section in the Materials and Methods and Table S4.

Table S1: Summary of community mean trait changes (per decade) across EUNIS Level 2 habitats. The table reports the linear slope estimates represent the interpolated mean change in values every 10 years across all plots of a given habitat. The color scheme for the slope column follows the categories defined in Figure 3 of the main manuscript. To ensure robust temporal coverage and limit extrapolation, interpolated time series for each habitat were restricted to plots sampled between the 0.05 and 0.95 quantiles of sampling years (\`start\` and \`end\` year, respectively).

EUNIS name	EUNIS code	Start year	End year	EIV	No. plots	CM_{EIV} /decade
Broadleaved deciduous forests	T1	1959	2016	Light	67,336	0.006
				Temperature	67,317	0.007
				Moisture	67,341	0.009
				Nitrogen	67,336	0.045
				Reaction	67,341	0.023
Broadleaved evergreen forests	T2	1974	2021	Light	3,940	-0.016
				Temperature	3,940	0.003
				Moisture	3,940	0.009
				Nitrogen	3,940	0.031
				Reaction	3,936	0.014
Coniferous forests	T3	1956	2016	Light	27,373	0.019
				Temperature	27,349	0.008
				Moisture	27,386	0.006
				Nitrogen	27,375	0.018
				Reaction	27,384	0.017
Dry grasslands	R1	1963	2018	Light	46,092	-0.024
				Temperature	46,241	-0.001
				Moisture	46,242	0.027
				Nitrogen	46,092	0.049
				Reaction	46,233	-0.011
Mesic grasslands	R2	1971	2017	Light	45,516	-0.017
				Temperature	45,519	0.004
				Moisture	45,520	-0.004
				Nitrogen	45,516	0.010
				Reaction	45,519	-0.011
Seasonally wet and wet grasslands	R3	1964	2017	Light	26,871	-0.016
				Temperature	26,871	0.001
				Moisture	26,873	-0.005
				Nitrogen	26,870	-0.010
				Reaction	26,871	-0.008
Alpine and subalpine grasslands	R4	1958	2017	Light	8,220	-0.022
				Temperature	8,395	0.014
				Moisture	8,508	0.002
				Nitrogen	8,211	0.010
				Reaction	8,395	-0.031
Woodland fringes and clearings and tall forb stands	R5	1969	2017	Light	13,526	-0.007
				Temperature	13,527	0.013

EUNIS name	EUNIS code	Start year	End year	EIV	No. plots	CM_{EIV} /decade
				Moisture	13,527	-0.011
				Nitrogen	13,526	-0.021
				Reaction	13,527	-0.007
Inland salt steppes and salt marshes	R6	1960	2021	Light	2,090	-0.021
				Temperature	2,093	-0.013
				Moisture	2,093	-0.013
				Nitrogen	2,090	0.037
				Reaction	2,093	-0.032
Tundra	S1	1965	2008	Light	759	0.001
				Temperature	759	0.004
				Moisture	759	-0.003
				Nitrogen	758	0.040
				Reaction	759	0.068
Arctic, alpine and subalpine scrub	S2	1959	2017	Light	4,473	0.003
				Temperature	4,515	0.017
				Moisture	4,541	-0.002
				Nitrogen	4,473	0.012
				Reaction	4,515	-0.014
Temperate and Mediterranean-montane scrub	S3	1967	2019	Light	6,176	0.000
				Temperature	6,192	0.004
				Moisture	6,196	0.010
				Nitrogen	6,176	0.029
				Reaction	6,196	-0.001
Temperate heathland	S4	1968	2019	Light	5,133	-0.019
				Temperature	5,131	0.002
				Moisture	5,133	0.011
				Nitrogen	5,133	0.015
				Reaction	5,133	-0.002
Maquis, arborescent matorral and thermo-Mediterranean scrub	S5	1973	2020	Light	2,095	-0.014
				Temperature	2,096	-0.006
				Moisture	2,096	0.024
				Nitrogen	2,094	0.079
				Reaction	2,093	0.009
Garrigue	S6	1956	2020	Light	717	-0.005
				Temperature	719	-0.005
				Moisture	711	0.016
				Nitrogen	716	0.052
				Reaction	718	-0.001
Spiny Mediterranean heaths	S7	1965	2010	Light	841	0.001
				Temperature	838	0.015
				Moisture	847	-0.004
				Nitrogen	839	0.026
				Reaction	848	0.021
Riverine and fen scrub	S9	1963	2018	Light	3,836	-0.013
				Temperature	3,836	0.008

EUNIS name	EUNIS code	Start year	End year	EIV	No. plots	CM_{EIV} /decade
				Moisture	3,838	-0.025
				Nitrogen	3,836	0.062
				Reaction	3,836	0.036
Raised and blanket bogs	Q1	1972	2022	Light	6,813	-0.013
				Temperature	6,809	0.009
				Moisture	6,813	-0.011
				Nitrogen	6,812	0.033
				Reaction	6,813	0.021
Valley mires, poor fens and transition mires	Q2	1960	2018	Light	9,378	-0.022
				Temperature	9,398	0.007
				Moisture	9,450	-0.034
				Nitrogen	9,377	0.014
				Reaction	9,397	0.006
Base-rich fens and calcareous spring mires	Q4	1997	2020	Light	33,002	-0.029
				Temperature	32,972	0.008
				Moisture	33,002	0.010
				Nitrogen	33,001	0.135
				Reaction	33,002	0.031
Helophyte beds	Q5	1963	2017	Light	28,462	-0.014
				Temperature	28,464	0.007
				Moisture	28,468	-0.061
				Nitrogen	28,458	0.036
				Reaction	28,466	-0.007
Periodically exposed shores	Q6	1954	2017	Light	6,104	-0.016
				Temperature	6,105	0.001
				Moisture	6,105	0.051
				Nitrogen	6,104	0.026
				Reaction	6,094	0.004

Table S2: Summary of plot observations and temporal coverage per analytical step. The table reports the number of plot observations and temporal coverage included in each analytical step of the study.

Methodological step	Dataset(s) used	Temporal coverage	No. plot observations	No. taxa available
Initial vegetation plot data before filtering (data in EVA/ReSurveyEurope proj. no. 222)	EVA + ReSurveyEurope	1873–2023	1,679,403	16,810
Final selection of vegetation plot data (after global filters)	EVA + ReSurveyEurope	1945–2023	692,393	13,874
Random Forests training (main models using broad, level-1 EUNIS habitat categories)	EVA + ReSurveyEurope	1945–2023	644,524 (= 622,906 EVA + 21,618 ReSurveyEurope; one randomly selected observation per time series)	13,874
Held-out ReSurveyEurope observations; randomly sampled for model validation	ReSurveyEurope	1945–2023	47,869	13,874
Random Forests training with EUNIS level-2 habitat	EVA + ReSurveyEurope subset with level-2 EUNIS	1945–2023	384,254	13,874
Prediction/interpolation set (level-1 EUNIS habitat categories)	EVA + ReSurveyEurope	1960–2020	610,537	13,874
Prediction/interpolation set (level-2 EUNIS habitat categories)	EVA + ReSurveyEurope subset with level-2 EUNIS	Depends on habitat type (see Table S1)	349,419	13,874
ReSurveyEurope-only RF validation (× 100 iterations)	ReSurveyEurope	1945–2023	21,618	13,874
Mixed-effects models	ReSurveyEurope	1960–2020	57,255 (from 18,345 resurvey plots)	13,874
Sensitivity analysis: CM_{EIV} vs CWM_{EIV} comparison (Random Forests and Mixed effect Models)	EVA + ReSurveyEurope plots with species cover data	1960–2020	477,990	13,874
Sensitivity analysis: CM_{EIV} excluding long-lived (tree) species (Random Forests)	Forest & Scrub only	1960–2020	221,710	13,029 (845 trees excluded)

Table S3: Cross-validation results for Random Forests models on training data. The table reports the results of Random Forests 10-fold cross-validation with three repeats obtained on the training data. *RMSE* = root mean squared error; *R-squared* = squared Pearson correlation of observed vs. predicted values $(\text{cor}_{\textit{obs vs. predicted}})^2$.

EIV	metric	mean	standard error
Light	RMSE	0.390	0.000396
	R-squared	0.885	0.000248
Temperature	RMSE	0.185	0.000234
	R-squared	0.932	0.000270
Moisture	RMSE	0.432	0.000471
	R-squared	0.864	0.000325
Nitrogen	RMSE	0.619	0.000681
	R-squared	0.784	0.000490
Reaction	RMSE	0.453	0.000458
	R-squared	0.860	0.000294

Table S4: Validation of EIV temporal interpolation tested on 21,826 resurvey plots available in the ReSurveyEurope dataset. Evaluation metrics are calculated on two sets of validation data: the change in community-mean indicator values within each time series (ΔCM_{EIV}) (see also Fig. S17) and the CM_{EIV} over 20% of the data saved for testing. *RMSE* = root mean squared error; R^2 = squared Pearson correlation of observed vs. predicted values $(cor_{obs\ vs.\ predicted})^2$; *cor* = correlation between observed and predicted values ($cor_{obs\ vs.\ predicted}$).

Validation test (predicted vs. observed)	Indicator	RMSE	R^2	cor
**ΔCM_{EIV}** (= $CM_{EIV\ end}$ - $CM_{EIV\ start}$)	Light	0.32 (SD: 0.002)	0.34 (SD: 0.0073)	0.58 (SD: 0.0062)
	Temperature	0.20 (SD: 0.0013)	0.26 (SD: 0.0095)	0.51 (SD: 0.0093)
	Moisture	0.32 (SD: 0.0026)	0.37 (SD: 0.0075)	0.61 (SD: 0.0061)
	Nitrogen	0.49 (SD: 0.0033)	0.26 (SD: 0.0078)	0.51 (SD: 0.0076)
	Reaction	0.42 (SD: 0.0034)	0.28 (SD: 0.0095)	0.53 (SD: 0.0089)
**Static CM_{EIV}** (20% testing data)	Light	0.33 (SD: 0.0072)	0.94 (SD: 0.0028)	0.97 (SD: 0.0014)
	Temperature	0.20 (SD: 0.0053)	0.92 (SD: 0.0049)	0.96 (SD: 0.0025)
	Moisture	0.35 (SD: 0.0073)	0.83 (SD: 0.0082)	0.91 (SD: 0.0045)
	Nitrogen	0.53 (SD: 0.0105)	0.83 (SD: 0.0066)	0.91 (SD: 0.0036)
	Reaction	0.45 (SD: 0.0103)	0.85 (SD: 0.0077)	0.92 (SD: 0.0042)
